# The Coppery Age: Copper (Cu)‐Involved Nanotheranostics

**DOI:** 10.1002/advs.202001549

**Published:** 2020-08-16

**Authors:** Caihong Dong, Wei Feng, Wenwen Xu, Luodan Yu, Huiijng Xiang, Yu Chen, Jianqiao Zhou

**Affiliations:** ^1^ Department of Ultrasound Zhongshan Hospital Fudan University Shanghai 200032 P. R. China; ^2^ School of Life Sciences Shanghai University Shanghai 200444 P. R. China; ^3^ State Key Laboratory of High Performance Ceramics and Superfine Microstructure Shanghai Institute of Ceramics Chinese Academy of Sciences Shanghai 200050 P. R. China; ^4^ Department of Ultrasound Ruijin Hospital Shanghai Jiaotong University School of Medicine Shanghai 200025 P. R. China

**Keywords:** antibacteria, bioimaging, cancer therapy, copper nanoparticles, nanomedicine, tissue regeneration

## Abstract

As an essential trace element in the human body, transitional metal copper (Cu) ions are the bioactive components within the body featuring dedicated biological effects such as promoting angiogenesis and influencing lipid/glucose metabolism. The recent substantial advances of nanotechnology and nanomedicine promote the emerging of distinctive Cu‐involved biomaterial nanoplatforms with intriguing theranostic performances in biomedicine, which are originated from the biological effects of Cu species and the physiochemical attributes of Cu‐composed nanoparticles. Based on the very‐recent significant progresses of Cu‐involved nanotheranostics, this work highlights and discusses the principles, progresses, and prospects on the elaborate design and rational construction of Cu‐composed functional nanoplatforms for a diverse array of biomedical applications, including photonic nanomedicine, catalytic nanotherapeutics, antibacteria, accelerated tissue regeneration, and bioimaging. The engineering of Cu‐based nanocomposites for synergistic nanotherapeutics is also exemplified, followed by revealing their intrinsic biological effects and biosafety for revolutionizing their clinical translation. Finally, the underlying critical concerns, unresolved hurdles, and future prospects on their clinical uses are analyzed and an outlook is provided. By entering the “Copper Age,” these Cu‐involved nanotherapeutic modalities are expected to find more broad biomedical applications in preclinical and clinical phases, despite the current research and developments still being in infancy.

## Graphical abstract

This work highlights the principles, progress, and prospects of the elaborate design and rational construction of Cu‐involved functional nanoplatforms for biomedical applications, including photonic nanomedicine, catalytic nanotherapeutics, antibacteria, accelerated tissue regeneration, and bioimaging. The engineering of Cu‐based nanocomposites for synergistic nanotherapeutics is also exemplified, followed by revealing their intrinsic biological effects and biosafety for revolutionizing their clinical translation.

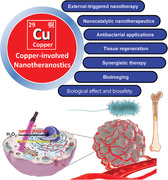

## 1 Introduction

The emerging of modern theranostic nanomedicine in the past decades has aroused broad research interests of the scientific community on exploring and developing versatile biomaterial nanosystems for satisfying the strict requirements of clinical medicine.^[^ ^1^, ^2^, ^3^, ^4^, ^5^, ^6^, ^7^ ^]^ This interdisciplinary field also promotes the concomitant development of new theranostic modalities on combating diverse diseases, where an overwhelming upsurge of nanomaterials is the fundamental/crucial basis and prerequisites determining the final theranostic performance.^[^ ^8^, ^9^, ^10^, ^11^, ^12^, ^13^ ^]^ Organic nanosystems have been extensively explored in nanomedicine, in accompany with clinically relevant family members entering the clinical stage.^[^ ^14^, ^15^, ^16^, ^17^, ^18^, ^19^, ^20^ ^]^ Comparatively, inorganic nanomaterials are mainly created in the past decade with noticeable advances very recently.^[^ ^21^, ^22^, ^23^, ^24^, ^25^ ^]^ These inorganic nanoplatforms feature inherent photonic, electronic, acoustic, and magnetic properties that are not possessed in traditional organic nanosystems.^[^ ^26^, ^27^, ^28^, ^29^ ^]^ Transitional metal element‐involved nanosystems are one of the most representative and exploited nanomaterials in theranostic nanomedicine, among which a myriad of iron (Fe)‐involved and manganese (Mn)‐composed nanosystems exert the specific function in disease treatment based on their magnetic/paramagnetic property, intrinsic microenvironment‐responsive behavior, and high biocompatibility, as reflected by thousands of publications regarding these two transitional metal element‐based biomaterial nanosystems.^[^ ^30^, ^31^, ^32^, ^33^, ^34^, ^35^ ^]^

Alternatively, copper ions (Cu^2+^) are the bioactive components with the specific capability for promoting angiogenesis, which is originated from their performance on stabilizing the expression of hypoxia‐inducible factor (HIF‐1*α*) and secretion of vascular endothelial growth factor (VEGF), further enhancing the recruitment and differentiation of cells during the blood vessel‐producing procedure.^[^ ^36^, ^37^, ^38^, ^39^, ^40^, ^41^, ^42^ ^]^ It has been fully revealed that Cu ions can facilitate the cell migration, angiogenesis, and collagen deposition for the specific biomedical implementation on accelerating wound healing.^[^ ^43^, ^44^, ^45^, ^46^ ^]^ As the necessary component for maintaining human health, the adults typically require the highest safe intake amount of Cu of 10 mg per day.^[^ ^47^ ^]^ As compared to Fe or Mn‐based nanosystems, Cu‐based nanoparticles feature their intrinsic physicochemical properties for satisfying varied biomedical application requirements, such as photothermal/photodynamic effects for photobased nanotherapy, catalytic activity for oxidative nanotherapy, specific interaction with drug molecules for chemotherapy, and antibacterial performances for combating bacterial infections. Therefore, it is reasonably anticipated that the construction of transitional metal Cu‐involved biomaterial nanosystems would generate a distinctive category of functional nanomaterials with unique theranostic performance in clinical medicine, originating not only from the biological effects of Cu ions but also from the physicochemical properties of the proposed Cu‐involved nanosystems.^[^ ^48^, ^49^ ^]^

On this ground, it has been well demonstrated that Cu‐based nanoagents feature photothermal‐conversion performance for near infrared (NIR)‐induced photonic tumor hyperthermia, especially including Cu‐based chalcogenides (e.g., CuS, Cu_9_S_5_, Cu_2−_ *_x_*Se).^[^ ^50^, ^51^, ^52^ ^]^ Especially, copper also possesses redox reactivity and easy replacement with other metals. Therefore, it can be developed as the catalyst for triggering specific catalytic reactions aiming to produce some reactive products for therapeutic purposes, such as the typical reactive oxygen species (ROS).^[^ ^53^ ^]^ Based on the advances of the nanosynthetic chemistry and material science, the morphology and nanostructure of Cu‐involved nanoplatforms can be precisely modulated and optimized for satisfying certain specific requirements, including the construction of a nanoporous structure and/or hollow nanostructure for efficient loading of therapeutic agents and drug delivery. Therefore, the structure/morphology modulation and physicochemical property manipulation of Cu‐involved nanosystems provide enormous potential and prospects for developing advanced theranostic nanomedicine.

**Figure 1:**
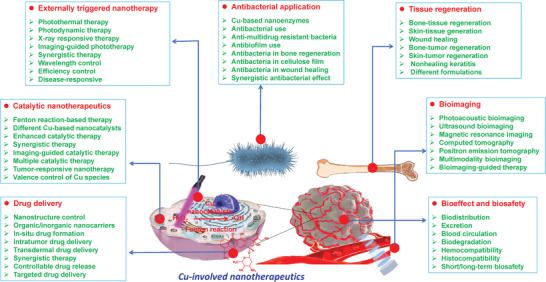
Summative scheme of the broad and versatile biomedical applications of Cu‐involved nanotherapeutics, predominantly including externally triggered nanotherapy, catalytic nanotherapeutics, drug delivery, antibacterial application, tissue regeneration, bioimaging, and bioeffects/biosafety. The main research aspects are included in each theranostic modality.

Considering the fast development of Cu‐involved nanosystems in theranostic nanomedicine very recently, we herein survey, summarize, and discuss the rational construction and versatile biomedical applications of Cu‐involved nanotherapeutics throughout this review (**Figure** **1**), including the design principles of these Cu nanoplatforms based on varied application purposes, the profound progresses of each Cu‐involved theranostic modalities and the critical issues hindering their further clinical translations. Numerous Cu‐composed nanosystems and their composites with abundant nanostructures and compositions have been constructed for versatile biomedical applications. This review initially clarifies their photonic property in biomedicine, including photothermal therapy (PTT) and photodynamic therapy (PDT), and the representative strategies/principles to strengthen the photonic performance by either nanoparticle design, external trigger variation, or response to inherent disease microenvironment. Then, the specific Cu nanocatalysts in catalytic medicine and corresponding nanotherapeutics have been highlighted in detail, regarding the intrinsic catalysis‐based therapeutic mechanism/principle and underlying strategies for enhancing the catalysis‐initiated nanotherapeutic efficacy. In addition to the most explored biomedical implementation in cancer theranostics, the antibacterial use and tissue regeneration of Cu‐involved nanotherapeutics are summarized and discussed in detail, in accompany with the development of Cu‐involved contrast agents for diagnostic bioimaging. The synergistic‐therapeutic modalities based on the elaborate design of Cu composite nanostructures are also clarified to achieve more efficient therapeutic consequences. The specific biological effects and biosafety of these Cu‐composed nanomaterials are also discussed to guarantee their further clinical translation (Table 1). Moving forward, the underlying critical issues, unresolved challenges, and future prospects are highlighted and an outlook is provided.

## 2 Cu‐Involved Nanoagents for Photonic Hyperthermia and Photodynamic Therapy

NIR is featured with less harmful effect, deeper tissue‐penetrating ability, and improved spatial/temporal tissue resolution as compared to traditional ultraviolet (UV) or visible light.^[^ ^54^, ^55^, ^56^, ^57^, ^58^, ^59^ ^]^ NIR‐induced PTT and PDT have developed very fast in the past decade based on the rapid progress of the emerging of versatile photothermal agents (PTAs) or photosensitizers (PSs) in nanoscale. The harnessing of PTAs is necessary because they can substantially augment the PTT efficacy, reduce the NIR power density and mitigate the damage of NIR to normal tissues. A multitude of metal‐based nanoagents have been explored for optical absorbance and PTT based on their tunable surface plasmon resonance (SPR) effect, such as the well‐known Au‐based nanoparticles with different morphologies and nanostructures.^[^ ^60^, ^61^, ^62^ ^]^ In addition, the nanosized photosensitizers can be activated by NIR for producing ROS and oxidative therapy.^[^ ^63^, ^64^, ^65^ ^]^ Fortunately, the rational engineering of Cu‐based nanosystems can achieve photonic nanotherapeutics with both photothermal and photodynamic effects.^[^ ^66^ ^]^

**Figure 2:**
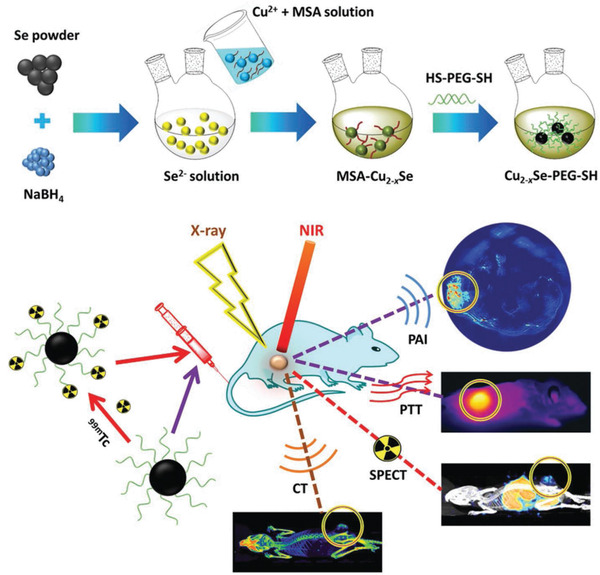
The scheme of the stepwise construction of Cu_2−_ *_x_*Se‐PEG‐SH nanoparticles and their specific functionality for photothermal ablation of tumor and CT/PA/SPECT imaging of tumor. Reproduced with permission.^[^ ^67^ ^]^ Copyright 2016, Wiley‐VCH.

Copper chalcogenides (Cu_2−_ *_x_*E, E: S, Se, Te, 0 ≤ *x* ≤ 1) have been extensively explored in photonic‐triggered disease treatment, such as photoacoustic (PA) imaging and photothermal hyperthermia.^[^ ^68^, ^69^ ^]^ Especially, cuprous sulfide with Cu‐deficient stoichiometries (Cu_2−_ *_x_*S) exhibited stoichiometry‐dependent localized surface plasmon resonance (LSPR) absorption in NIR range and photothermal conversion. The LSPR effect in novel metals predominantly originates from the oscillation of free electrons. Comparatively, the LSPR effect of copper chalcogenides is dominantly attributed to free holes originating from cation vacancies.^[^ ^66^ ^]^ An ambient aqueous synthetic strategy was developed for the fabrication of ultrasmall PEGylated Cu_2−_ *_x_*Se nanoparticles for photothermal tumor ablation.^[^ ^67^ ^]^ The ultrasmall size (around 3.6 nm) was chosen because of the prolonged blood‐circulation duration with less clearance by phagocytes and reticuloendothelial system (RES) and enhanced tumor accumulation by the typical enhanced permeability and retention (EPR) effect. The surface PEGylation with ‐SH group provided the anchoring sites to label technetium‐99m (^99m^Tc) for single‐photon emission computed tomography (SPECT) imaging (**Figure** **2**). The PEGylated Cu_2−_ *_x_*Se nanoparticles were featured with high extinction coefficient of 8.5 L g^−1^ cm^−1^ and photothermal‐conversion efficiency of 64.8% at 808 nm. Based on the desirable photothermal conversion of PEGylated Cu_2−_ *_x_*Se nanoparticles, the tumor growth was substantially inhibited with no reoccurrence and metastasis. Their multimodality imaging capability (computed tomography (CT), PA, and SPECT) might provide the imaging guidance or monitoring for nanotherapeutics.^[^ ^67^ ^]^ Similarly, ferritin (Fn) nanocages were harnessed to synthesize ultrasmall copper sulfide (CuS) nanoparticles based on a biomimetic procedure for photothermal nanotherapeutics of tumors with PA and positron emission tomography (PET) dual‐modality bioimaging guidance.^[^ ^70^ ^]^

Rattle‐type Fe_3_O_4_@CuS composite nanoparticles were rationally constructed to solve the critical issue of tumor targeting and suitable NIR wavelength.^[^ ^71^ ^]^ The integration of magnetic Fe_3_O_4_ nanoparticles exerted the functionality of magnetic targeting for strengthening the tumor accumulation. Importantly, the photonic response in the second NIR biowindow (1064 nm) of these Fe_3_O_4_@CuS composite nanoparticles achieved higher tissue‐penetrating capability, inducing enhanced tumor‐inhibiting rate with no further relapse as compared to the laser activation by the first NIR biowindow (808 nm). In addition to the hydrophilic plate‐like Cu_9_S_5_ nanocrystals with improved absorption (1.2 × 10^9^ m ^−1^ cm^−1^) and photothermal‐conversion efficiency (25.7%) at 980 nm,^[^ ^72^ ^]^ hydrophilic flower‐like CuS superstructures were exemplified to respond to external 980 nm laser activation for achieving photothermal conversion and the following ablation of cancer cells.^[^ ^73^ ^]^ Cysteine‐coated CuS nanoparticles were also irradiated by 980 nm laser with high photothermal‐conversion efficiency of 38.0% where the tumor growth was significantly inhibited at the low CuS dose and safe 980 nm laser power density of 0.72 W cm^−2^.^[^ ^74^ ^]^ In addition, the encapsulation of CuS nanoparticles into zeolitic imidazole framework‐8 (ZIF‐8) caused the NIR‐induced dissociation of ZIF‐8 for the release of the loaded chemotherapeutic drug, aiming to achieve synergistic photothermal ablation and NIR‐triggered chemotherapy.^[^ ^75^ ^]^ The doping of magnetic ferric ions (Fe^3+^) modulated the vacancy of Cu_2−_ *_x_*Se nanoparticles for manipulating the NIR absorption, which also endowed these semiconductors with MR imaging performance.^[^ ^76^ ^]^ To improve the photothermal‐conversion efficiency, Cu_2−_ *_x_*S and Ag_2_S were integrated into one system by producing Cu–Ag_2_S/PVP nanoparticles with high photothermal‐conversion efficiency of 58.2% under 808 nm laser irradiation, much higher as compared to that of Cu_2−_ *_x_*S/PVP nanoparticles (27.1%).^[^ ^77^ ^]^

**Figure 3:**
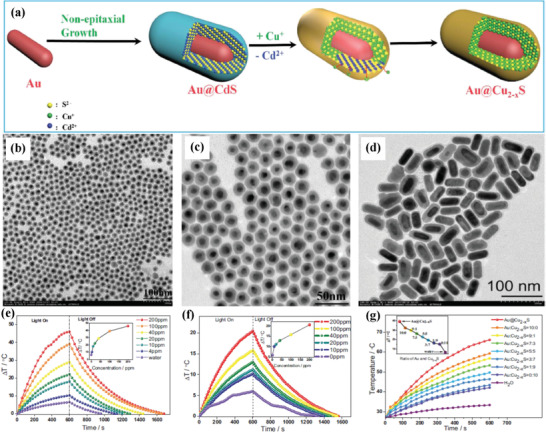
a) Schematic illustration on the construction of Au@Cu_2−_ *_x_*S core/shell nanoparticles by a cation‐exchange method between Au@CdS nanoparticles and Cu^+^. TEM images of Au@Cu_2−_ *_x_*S nanoparticles in the form of b,c) nanoparticles and d) nanorods. Temperature evaluation of Au@Cu_2−_ *_x_*S nanorods aqueous colloidal solution by e) 808 nm laser (1 W cm^−2^) and f) 1064 nm laser (0.7 W cm^−2^) irradiations. g) The photothermal‐performance comparison of Au@Cu_2−_ *_x_*S and the physical mixture of Au nanorods and Cu_2−_ *_x_*S nanoparticles at different ratios. Reproduced with permission.^[^ ^78^ ^]^ Copyright 2016, Wiley‐VCH.

The rational integration of plasmonic Au nanoparticles and plasmonic Cu_2−_ *_x_*S semiconductor into one matrix could enhance the photothermal performance of either Au or Cu_2−_ *_x_*S component. The coupled LSPR properties of Au and Cu_2−_ *_x_*S could be maximized by the design of Au@Cu_2−_ *_x_*S core/shell nanoparticles for augmented PTT efficacy. Au@CuS nanoparticles were initially synthesized with the following cation exchange between Cu^+^ and CdS shell to produce Au@Cu_2−_ *_x_*S nanostructures (**Figure** **3**a), which could be tuned with high dispersity in the form of either nanoparticles (Figure 3b,c) or nanorods (Figure 3d).^[^ ^78^ ^]^ The corresponding photothermal‐conversion efficiency was calculated to be 59% at 808 nm and 43% at 1064 nm, which quickly elevated the surrounding temperature of Au@Cu_2−_ *_x_*S nanorods aqueous solution (Figure 3e,f). Especially, the design of core/shell Au@Cu_2−_ *_x_*S was more favorable as compared to the simple mixture of Au nanorods and Cu_2−_ *_x_*S nanoparticles for photothermal conversion (Figure 3e). Such a core/shell design with improved photothermal performance also induced more HeLa cell death as compared to Cu_2−_ *_x_*S at the equivalent concentration. An Au–Cu_9_S_5_ plasmonic hybrid nanosystem was constructed for enhancing the LSPR of Cu_9_S_5_ by the coupling effect of LSPR, which was based on the collective oscillations of electron and hole.^[^ ^79^ ^]^ This Au–Cu_9_S_5_ hybrid nanosystem exhibited enhanced absorption cross‐section of 1.3 × 10^8^ m ^−1^ cm^−1^ and high photothermal transduction efficiency of 37% in the second NIR biowindow (1064 nm), efficiently exerting the photothermal ablation of tumor tissue in NIR‐II biowindow. Similarly, spherical Au@Cu_2−_ *_x_*S, Au@Cu_2−_ *_x_*Se and rod‐like Au@Cu_2−_ *_x_*S supraparticles were synthesized for photothermal ablation of tumors (4T1 tumor model) based on the plasmonic coupling effect between the core and shell, in accompany with the X‐ray computed tomography imaging capability because of the presence of Au component with large atomic number and X‐ray attenuation coefficient (5.16 cm^−2^ kg^−1^).^[^ ^80^ ^]^

**Figure 4:**
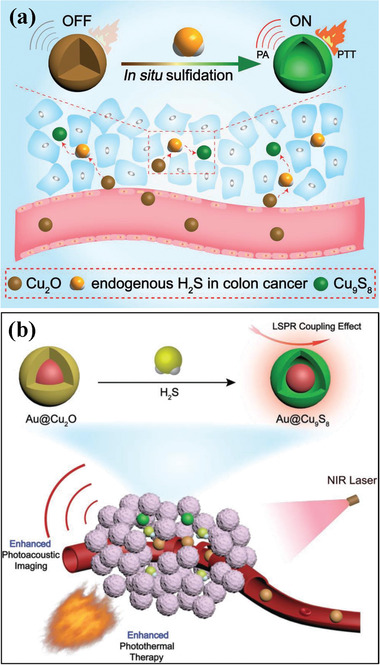
a) Schematic illustration of the conversion of Cu_2_O into Cu_9_S_8_ nanoparticles by endogenous H_2_S‐based in situ reaction, enabling the PA imaging and photothermal ablation of colon cancer. Reproduced with permission.^[^ ^81^ ^]^ Copyright 2018, Wiley‐VCH. b) Schematic illustration of the conversion of Au@Cu_2_O into Au@Cu_9_S_8_ by in situ H_2_S reaction for contrast‐enhanced PA imaging of photothermal hyperthermia of colon cancer with the performance enhancement by the LSPR coupling effect. Reproduced with permission.^[^ ^82^ ^]^ Copyright 2019, Wiley‐VCH.

In addition to the general features of tumor microenvironment (TME) for nanotherapeutics such as the mild acidity,^[^ ^83^, ^84^, ^85^ ^]^ reducing condition,^[^ ^86^, ^87^ ^]^ and hypoxia,^[^ ^88^, ^89^, ^90^ ^]^ some tumor types have their inherent characteristics for providing the foundation on nanomedicine design. Based on the fact that the upregulation of hydrogen sulfide (H_2_S)‐generating enzyme of cystathionine‐*β*‐synthase (CBS) in colon cancer, the H_2_S concentration in tumor reaches around 0.3 to 3.4 mmol L^−1^. Therefore, this overexpressed endogenous H_2_S was harnessed for in situ conversion of cuprous oxide (Cu_2_O) into copper sulfide for activatable PA imaging and photothermal tumor ablation (**Figure** **4**a).^[^ ^81^ ^]^ It was exemplified that the implementation of S‐adenosyl‐l‐methionine (SAM) as an allosteric CBS activator expedited the in situ reaction between H_2_S and Cu_2_O, thus producing significantly strengthened PA imaging signals and photothermal effects for elevating the tumor temperature after 808 nm laser irradiation. Comparatively, the use of aminooxy‐acetic acid (AOAA) as the CBS inhibitor lowered the H_2_S production and subsequently decreased the conversion of Cu_2_O into copper sulfide, exhibiting no obvious PA signal in tumor and negligible temperature variation. However, the photothermal‐conversion efficiency of converted copper sulfide was low with a high dose to achieve desirable photonic therapeutic consequence. To solve this critical issue, the Au@Cu_2_O plasmonic hybrids were constructed for enhanced photothermal performance after in situ H_2_S‐triggered conversion based on the coupling effect of LSPR between noble metal and plasmonic semiconductor (Figure 4b).^[^ ^82^ ^]^ Similar to the conversion of Cu_2_O into Cu_9_S_8_, the tumor‐accumulated Au@Cu_2_O nanoparticles were also converted into Au@Cu_9_S_8_ nanoagents for achieving contrast‐enhanced PA imaging and photothermal tumor ablation by elevating the tumor temperature. The LSPR coupling effect induced nearly 2.1 times of stronger NIR absorption and 1.2 times of photothermal‐conversion efficiency elevation, resulting in the harnessing of low nanoparticle dose with the desirable theranostic performance. These two paradigms provide an alternative strategy for achieving photothermal hyperthermia of Cu‐involved nanoagents by in situ generation of Cu‐based nanoagents with distinctive photothermal performance.

**Figure 5:**
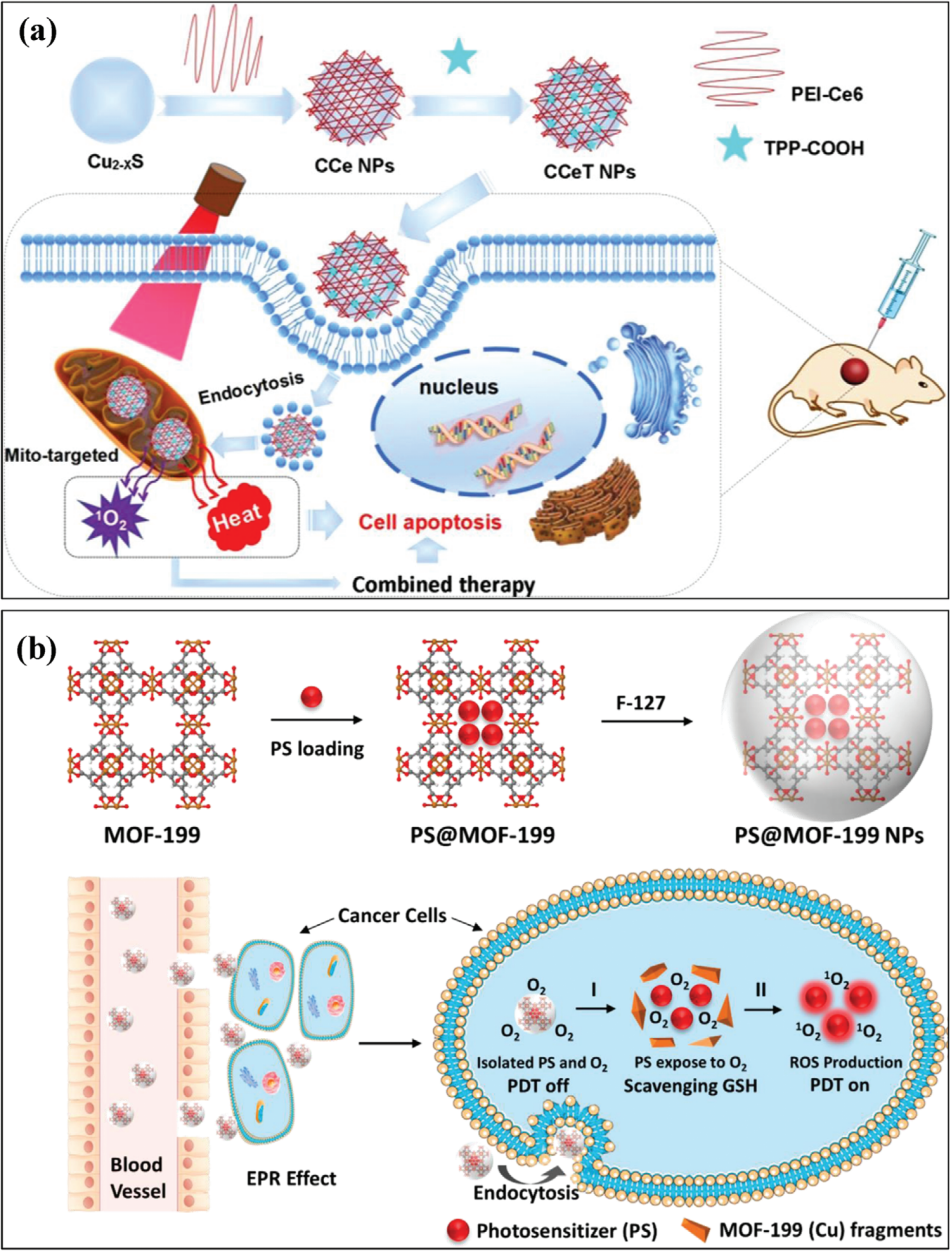
a) Schematic illustration on the construction of CCeT nanoparticles and the underlying therapeutic mechanism on mitochondria‐targeted and NIR‐activated synergistic photothermal ablation and PDT. Reproduced with permission.^[^ ^91^ ^]^ Copyright 2018, American Chemical Society. b) The scheme on the fabrication of PS@MOF‐199 and F127‐coated PS@MOF‐199 nanoparticles, and their therapeutic mechanism involving the accumulation into tumor via EPR effect, TME‐sensitive MOF disintegration by the interaction between Cu(II) and GSH, and enhanced singlet oxygen (^1^O_2_) production by external light activation for inducing PDT effect. Reproduced with permission.^[^ ^92^ ^]^ Copyright 2019, American Chemical Society.

Based on the inherent photothermal effect of Cu_2−_ *_x_*S nanoparticles, the endowed photodynamic effect was accomplished by rational integration of Cu_2−_ *_x_*S nanoparticles with photosensitizer Ce6‐conjugated branched polyethyleneimine.^[^ ^91^ ^]^ The targeted delivery was enabled by surface conjugation of mitochondria‐targeted molecule TPP‐COOH (abbreviated as CCeT nanoparticles). The mitochondrial‐targeted delivery and synergistic PTT/PDT (**Figure** **5**a) almost completely eradicated the tumor in vivo by both 630 nm laser for activating Ce6 photosensitizer (PDT) and 808 nm laser for stimulating Cu_2−_ *_x_*S nanoparticles (PTT). The PS with aggregation‐induced emission property (2‐(4‐(diphenylamino)phenyl)anthracene‐9,10‐dione) was loaded into a Cu(II) carboxylate metal–organic framework (MOF) (MOF‐199, HKUST‐1) for glutathione (GSH) consuming‐enhanced PDT (Figure 5b).^[^ ^92^ ^]^ The reaction between Cu(II) component with intracellular GSH would induce the MOF dissociation and trigger the PSs release, which was activated by external light activation for inducing PDT effect with enhanced efficiency because of GSH consumption. Similarly, Cu(II)‐metalated nano‐MOF with Cu(II) as the active center for binding/absorption of GSH and porphyrin ligand as the photosensitizer component was fabricated to achieve strengthened PDT efficacy, which showed comparable therapeutic consequence as compared to the typical anticancer drug camptothecin.^[^ ^92^ ^]^ As the degenerately doped semiconductors, Cu_2−_ *_x_*S nanoparticles themselves were also exemplified to respond to external NIR irradiation for producing ROS such as singlet oxygen (^1^O_2_) and hydroxyl (•OH) radicals.^[^ ^93^ ^]^ Therefore, these Cu_2−_ *_x_*S nanoparticles were developed as photosensitizers for PDT, in combination with photothermal effect to induce synergistic PDT/PTT tumor therapy.

**Figure 6:**
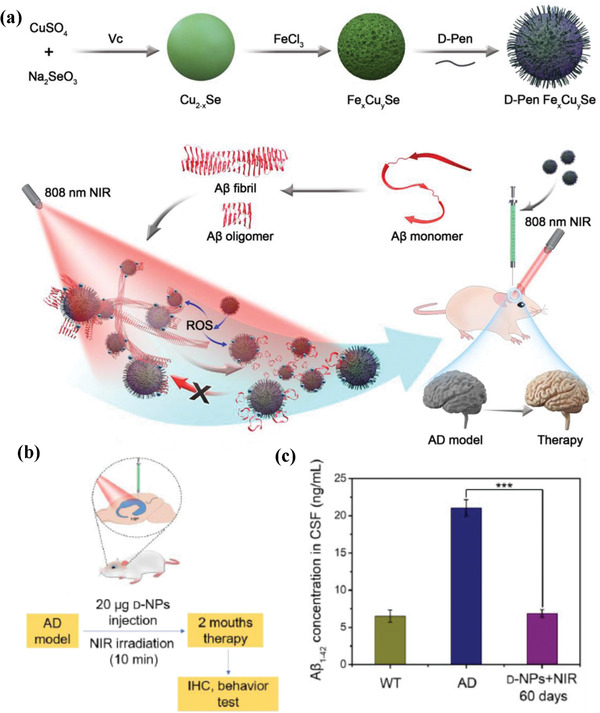
a) Schematic illustration on the construction of penicillamine‐modified Fe*_x_*Cu*_y_*Se nanoparticles, and the underlying mechanism of 808 nm laser‐induced inhibition and disassembly of d‐Pen Fe*_x_*Cu*_y_*Se on A*β*42 aggregation, resulting in the alleviation of neurotoxicity in AD mice model. b) In vivo animal experiment on the administration of d‐Pen Fe*_x_*Cu*_y_*Se nanoparticles into AD mice model, and c) the A*β*42 concentration in cerebrospinal fluid following the 60 days treatment. Reproduced with permission.^[^ ^95^ ^]^ Copyright 2020, Wiley‐VCH.

In addition to the photonic nanomedicine on tumor nanotherapeutics, the photoresponse of Cu‐involved nanoparticles can be developed for other versatile biomedical uses. It has been demonstrated that Alzheimer's disease (AD) is correlated to the accumulation and deposition of *β*‐amyloid (A*β*) plaques in thebrain. Based on this fact, chiral l/d‐Fe*_x_*Cu*_y_*Se nanoparticles (abbreviated as l/d‐NPs) were engineering for responding to 808 nm laser irradiation and producing ROS (single oxygen and hydroxyl radicals) without photothermal effect, which converted the dense structure of A*β*42 fibrils into looser monomers (**Figure** **6**a).^[^ ^95^ ^]^ Especially, the d‐Fe*_x_*Cu*_y_*Se nanoparticles exhibited an enhanced affinity for A*β*42 fibrils as compared to either l‐Fe*_x_*Cu*_y_*Se or chiral Cu_2−_ *_x_*Se nanoparticles. Based on the injection of d‐Fe*_x_*Cu*_y_*Se nanoparticles into the brains of APP/PS1 transgenic mouse model followed by NIR irradiation each day in 60 days (Figure 6b), the in vivo experiment signified that they significantly decreased the A*β*42 concentration in the AD mice within two months (Figure 6c). These d‐Fe*_x_*Cu*_y_*Se nanoparticles also protected the A*β*42‐induced neuronal damage and mitigated the symptoms in AD mouse model.

Despite Cu‐involved nanosystems that can respond to light activation for either photothermal hyperthermia or photodynamic interaction, the intrinsic low tissue‐penetrating ability of light limits their applications in the treatment of superficial diseases. Therefore, the design of photoresponsive Cu‐involved nanoagents should be focused on their design/construction with high photothermal‐conversion efficiency and prolonged photoresponsive wavelength of light (typically in the range of NIR‐II), which can partially address this critical issue. It is noted that the photothermal or photodynamic effects of certain Cu‐based nanoparticles strongly depend on the nanostructure, composition, and Cu valences of these nanosystems. The therapeutic outcome and potential clinical translation are highly related to practical application requirements. In addition, the development of light‐free therapeutic modalities with the participation of Cu‐involved nanoagents has become one of the intriguing research frontiers, including the following discussed applications in catalytic medicine by taking the features of catalytic activities of these Cu‐involved nanocatalysts.

## 3 Cu‐Involved Nanocatalysts for Catalytic Medicine

Catalytic medicine based on the creation of diverse nanocatalysts on triggering specific chemical reactions in diseases has emerged as a distinctive therapeutic modality with high disease specificity and low side effects.^[^ ^10^, ^96^, ^97^, ^98^ ^]^ Especially, catalytic Fenton reaction‐based nanotherapeutics depend on the generation of toxic ROS by converting hydrogen peroxide (H_2_O_2_) into hydroxyl radicals under the mildly acidic condition.^[^ ^99^, ^100^, ^101^, ^102^, ^103^, ^104^, ^105^ ^]^ Traditional Fe‐based Fenton nanoagents suffer from the low effective pH‐operating condition (pH = 3–4) and slow reaction rate (≈63 m ^−1^ s^−1^). Comparatively, Cu‐base Fenton nanoagents are featured with more adaptable reactive pH ranges with high Fenton reaction rate (≈1 × 10^4^ m ^−1^ s^−1^), which can also convert H_2_O_2_ into toxic hydroxyl radicals.^[^ ^53^ ^]^ Therefore, diverse Cu‐based Fenton nanoagents or their composites have been developed for oxidative tumor therapy. For instance, Cu‐amino acid mercaptide (Cu‐Cys) nanoparticles were designed to achieve GSH‐responsive chemodynamic tumor therapy, where the in situ GSH in tumor reduced Cu^2+^ into Cu^+^ for effectively converting H_2_O_2_ into hydroxyl radicals by catalytic Fenton reaction, which subsequently induced cancer‐cell death and tumor‐growth inhibition.^[^ ^106^ ^]^

**Figure 7:**
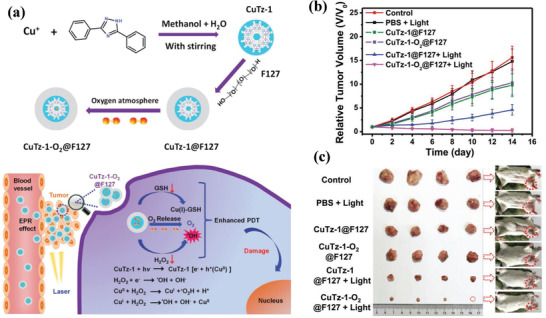
a) Schematic illustration on the construction of CuTz‐1‐O_2_@F127 nanoparticles and their specific light‐triggered reaction intracellularly for the simultaneous production of hydroxyl radicals (•OH) and singlet oxygen (^1^O_2_). b) The relative tumor‐volume changes of tumor‐bearing mice after varied treatments including PBS, PBS + light, CuTz‐1@F127, CuTz‐1‐O_2_@F127, CuTz‐1@F127 + light, and CuTz‐1‐O_2_@F127 + light. c) The corresponding photographic image of excised tumors in different treatment groups. Reproduced with permission.^[^ ^107^ ^]^ Copyright 2019, Wiley‐VCH.

O_2_‐loaded CuTz‐1@F127 MOF (abbreviated as CuTz‐1‐O_2_@F127; **Figure** **7**a) was constructed for the simultaneous production of two kinds of ROS radicals, including Fenton‐like reaction‐based hydroxyl radical (•OH) and photodynamic effect‐induced singlet oxygen (^1^O_2_).^[^ ^107^ ^]^ In addition, the oxygen loading and delivery significantly alleviated the tumor hypoxia and the released Cu^2+^ consumed intracellular GSH, both of which further strengthened the efficacy of oxidative cancer therapy. Based on the in vivo 4T1 tumor‐bearing female BALB/c mice model, the therapeutic evaluation signified that the combined effect of O_2_ delivery into the tumor, light‐triggered dual radical production and facile biodegradation of the nanocarriers achieved both high tumor‐suppression rate and therapeutic biosafety (Figure 7b,c). To solve the critical issue of low H_2_O_2_ amount in the tumor microenvironment during Fenton reaction, copper peroxide nanodots were designed with the specific functionality of initiating H_2_O_2_ self‐supplying catalytic Fenton reaction.^[^ ^108^ ^]^ The copper peroxide initially reacted with H_2_O to generate H_2_O_2_. The tumor acidic condition decomposed copper peroxide to release Cu ions, which acted as the catalysts for converting self‐supplying H_2_O_2_ into highly toxic hydroxyl radicals and consequently inhibiting tumor growth.^[^ ^108^ ^]^ Similarly, the co‐attachment of superoxide dismutase (SOD) and Cu component into calcium carbonate (CaCO_3_)‐mineralized nanoparticles also achieved the production of hydrogen peroxide by SOD and efficient Fenton‐like reaction as catalyzed by the doped Cu component, resulting in the hydroxyl radicals production and specific toxicity to cancer cells.^[^ ^109^ ^]^

**Figure 8:**
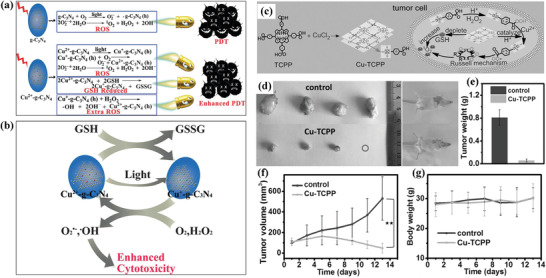
a) Schematic illustration of the underlying chemical reactions of g‐C_3_N_4_ nanosheets for producing photodynamic effect, and Cu^2+^‐C_3_N_4_ nanosheets for enhancing the photodynamic effect and inducing synergistic effect on supplementary ROS production and GSH depletion. b) The scheme of a detailed procedure of Cu^2+^‐C_3_N_4_ nanosheets for enhanced PDT by improving ROS generation and reducing GSH level. Reproduced with permission.^[^ ^110^ ^]^ Copyright 2016, Wiley‐VCH. c) Synthetic scheme of Cu‐TCPP nanosheets and the intrinsic therapeutic mechanism. d) Photographic image of mice and excised tumors at the end of treatment. e) Tumor weight and f) tumor‐volume changes with time after the treatments. g) The corresponding body‐weight changes with time. Reproduced with permission.^[^ ^111^ ^]^ Copyright 2019, Wiley‐VCH.

Based on the photodynamic effect of 2D graphitic carbon nitride (g‐C_3_N_4_) as photosensitizers for producing ROS, Cu^2+^ was coordinated with g‐C_3_N_4_ (designated as Cu^2+^‐C_3_N_4_) for further enhancing the PDT effect based on two contributions.^[^ ^110^ ^]^ On one hand, Cu^2+^ in Cu^2+^‐C_3_N_4_ was reduced by intracellular GSH to generate Cu^+^, which also consumed GSH for further reducing the PDT‐induced ROS depletion because of the reducing effect of GSH (**Figure** **8**a). On the other hand, the postgenerated Cu^+^ not only transformed molecular oxygen to superoxide anion, but also converted H_2_O_2_ into hydroxyl radicals by catalytic chemical reactions (Figure 8b). This work provides a specific strategy to enhance g‐C_3_N_4_‐based PDT efficacy by copper(II) coordination and the inherent catalytic performance of Cu ions.^[^ ^110^ ^]^ 2D Cu‐TCPP (TCPP: tetrakis(4‐carboxyphenyl)porphyrin) nanosheets with metal–organic framework were fabricated to produce singlet oxygen (^1^O_2_) in hypoxia tumor by the Russell mechanism.^[^ ^111^ ^]^ The peroxidation of TCPP ligand was enabled by H_2_O_2_ in acidic TME condition, which was further reduced to ROO^•^ radicals by the action of Cu‐TCPP nanosheets with peroxidase‐mimicking property and Cu^2+^ ions. The spontaneous recombination of ROO^•^ generated ^1^O_2_ for killing cancer cells based on the underlying Russell mechanism (Figure 8c). The intravenous administration of Cu‐TCPP nanosheets significantly suppressed the tumor growth (Figure 8d–f) on tumor‐bearing Kunming mice with negligible body‐weight change (Figure 8g). This work regarding Cu‐TCPP achieves ^1^O_2_ production independent on the oxygen level and external light irradiation, therefore it is anticipated to overcome some critical issues of traditional PDT.

**Figure 9:**
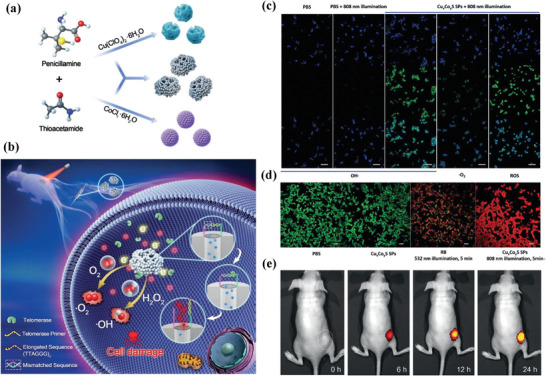
a) Schematic illustration of the fabrication of Cu*_x_*Co*_y_*S SPs and b) the underlying mechanism of Cu*_x_*Co*_y_*S‐enabled telomerase‐responsive bioimaging and oxidative cell damage by produced ROS. c) Fluorescent imaging of MCF‐7 cancer cells for showing the intracellular ROS production with green fluorescence. d) Fluorescent imaging of live (green fluorescence) and dead (red fluorescence) cells. e) Time‐dependent fluorescent imaging of tumor xenograft after the administration of Cy5.5‐loaded Cu*_x_*Co*_y_*S SPs. Reproduced with permission.^[^ ^112^ ^]^ Copyright 2019, Wiley‐VCH.

Cu*_x_*Co*_y_*S superaparticles (SPs) were fabricated for NIR‐induced photocatalysis on ROS production, which was fabricated by a specific spontaneous assembly process (**Figure** **9**a).^[^ ^112^ ^]^ After the attachment of cyanine5.5 (Cy5.5) into the pores of these Cu*_x_*Co*_y_*S SPs with the surface locking by complementary DNA sequence, the intracellular telomerase would react with the surface‐locked DNA to trigger the release of Cy5.5 from the pores of Cu*_x_*Co*_y_*S SPs, inducing the recovery of Cy5.5 fluorescence for telomerase‐sensitive bioimaging (Figure 9b). The phototriggered intracellular large production of ROS (Figure 9c) was the main form of hydroxyl radical (•OH), which significantly induced MCF‐7 cancer‐cell death (Figure 9d). The in vivo bioimaging experiment also demonstrated the strong fluorescence on tumor xenograft after the administration of Cy5.5‐loaded Cu*_x_*Co*_y_*S SPs (Figure 9e). Indocyanine green (ICG)‐loaded Cu^2+^‐protein self‐assemblies played the catalytic role on converting H_2_O_2_ into toxic hydroxyl radicals. Meanwhile, this nanocomposite expedited oxygen production because of its catalase activity, further strengthening the PDT efficacy of loaded ICG.^[^ ^113^ ^]^ The highly active (102) surface of biodegradable CuS nanoparticles (containing Cu^+^) was exemplified to be easily degraded by pH and laser irradiation at 808 nm, which released large amounts of Cu^+^ for producing sufficient ROS by laser‐enhanced catalytic Fenton‐like chemical reaction with desirable tumor‐therapeutic consequence.^[^ ^114^ ^]^

**Figure 10:**
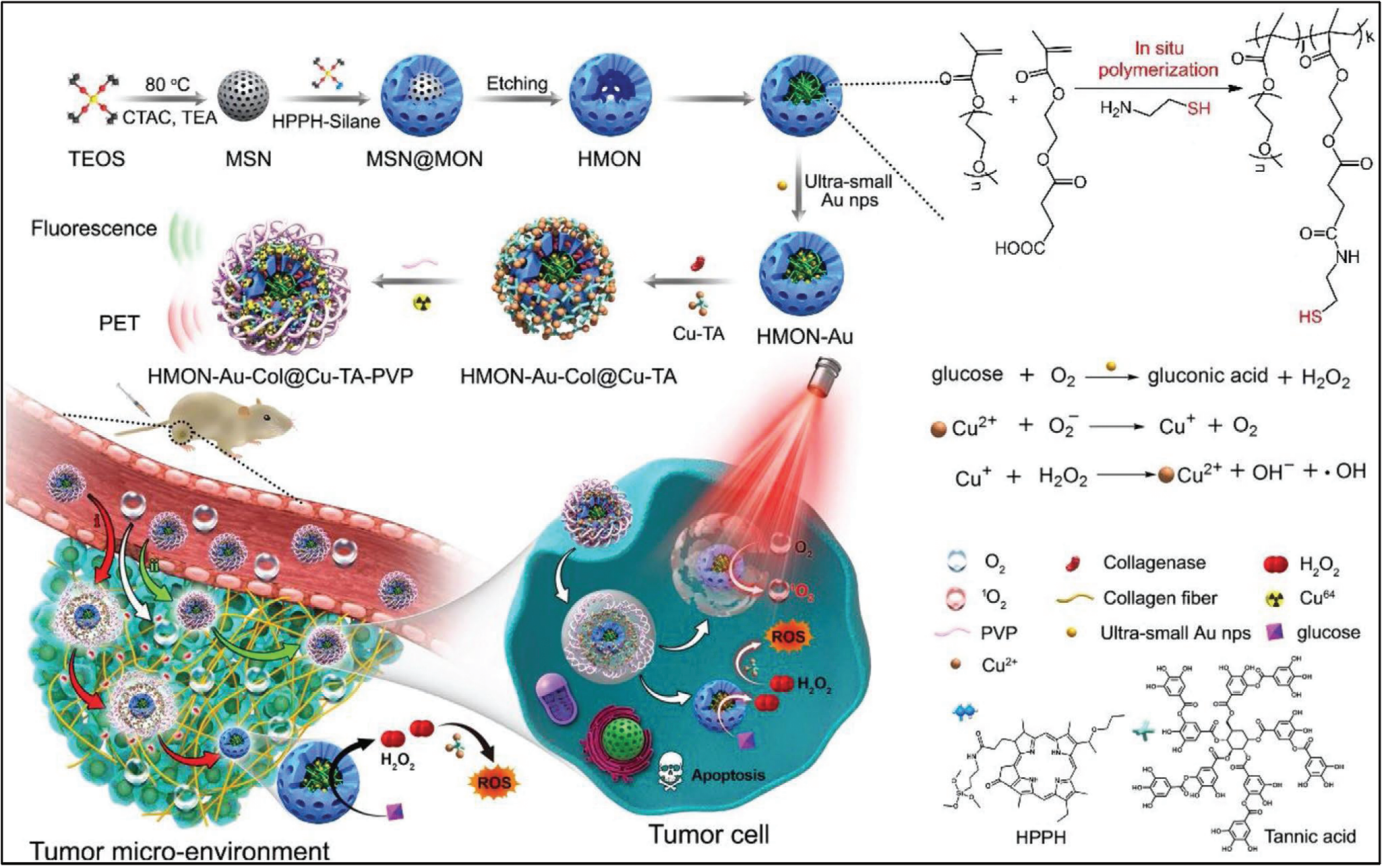
Schematic illustration on the construction of HMON‐Au‐Col@Cu‐TA‐PVP composite nanoreactor for synergistic exogenously responsive PDT and endogenously responsive CDT against tumors. Reproduced with permission.^[^ ^115^ ^]^ Copyright 2020, Wiley‐VCH.

Mesoporous organosilica nanoparticles (HMONs) were used as the substance for the integration of photodynamic modality and chemodynamic modality to achieve synergistic cancer therapy (**Figure** **10**). The photosensitizer was directly hybridized into the framework of HMONs to avoid the blocking of mesopores. The Au nanoparticles were loaded into the large hollow interior by using thiol groups of a preloaded polymer, which acted as the glucose oxidase‐mimicking nanoenzyme to produce hydrogen peroxide (H_2_O_2_). Cu^2+^‐tannic acid complex was modified onto the surface of HMONs for triggering Fenton‐like catalytic reaction using pregenerated H_2_O_2_ as the reactant. Therefore, the final constructed HMON‐Au‐Col@Cu‐TA‐PVP composite nanoreactor featured synergistic functionality of photonic PDT and chemodynamic therapy (CDT) for achieving the highest tumor‐suppressing efficacy as compared to either PDT or CDT single therapeutic modality.^[^ ^115^ ^]^ Cu^2+^ was hybridized into the framework of large pore‐sized mesoporous silica with doxorubicin (DOX) loading for triggering ROS production and chemotherapy, resulting in enhanced intracellular oxidative stress and immunogenic cell death. Accordingly, this nanosystem acted as the immune adjuvants to stimulate the immune‐cell maturation and induced the synergistic efficacy on PD‐L1 antibody‐based immunotherapy, not only resulting in the suppression of treated primary tumors but also inducing the inhibition of nontreated distant tumors.^[^ ^116^ ^]^

**Figure 11:**
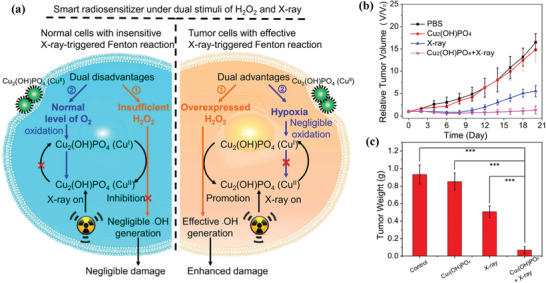
a) The scheme of the reaction mechanism of X‐ray‐induced Fenton reaction as enabled by Cu_2_(OH)PO_4_@PAAS NCs in either normal cells with insensitive X‐ray‐activated Fenton reaction or tumor cells with effective X‐ray‐triggered Fenton reaction. b) Relative tumor‐volume changes as a function of treatment duration after varied treatments as shown in the figure, and c) corresponding tumor weight after the treatment for 20 days. Reproduced with permission.^[^ ^94^ ^]^ Copyright 2019, American Chemical Society.

In addition to the most explored tumor microenvironment‐activated Fenton reaction for tumor‐oxidative therapy by Cu‐involved nanocatalysts, the external X‐ray as the stimulus was employed for triggering Cu‐based Fenton reaction and subsequent tumor‐oxidative therapy.^[^ ^94^ ^]^ In detail, copper hydroxyphosphate nanocatalysts (Cu_2_(OH)PO_4_ NC) were initially synthesized followed by further surface mediation with poly(acrylic acid) sodium (PAAS). The exogenous X‐ray irradiation converted Cu^II^ sites into Cu^I^ sites, which was more catalytically active in triggering Fenton reaction (**Figure** **11**a). The postgenerated Cu^I^ sites further converted tumor‐overexpressed H_2_O_2_ into hydroxyl radicals for inducing cancer cell death. Comparatively, the normal tissue with high oxygen level but low H_2_O_2_ amount could not initiate the Fenton reaction for producing toxic hydroxyl radicals. A more obvious tumor‐suppression efficacy was achieved by the combinatorial use of Cu_2_(OH)PO_4_ NC and X‐ray activation (Figure 11b,c), signifying the desirable externally triggered Fenton reaction‐based high‐efficient tumor‐oxidative therapy. This paradigm designed Cu‐based nanoparticles as both radiosensitizer and nanocatalyst for externally responsive tumor nanotherapeutics.

The design principle of Cu‐involved nanocatalysts in Fenton‐like catalytic reaction gives consideration to two aspects. The first priority is the high catalytic performance, where the precise modulation of Cu valence (e.g., Cu^+^ amount) and the reactant amount (e.g., H_2_O_2_ amount) plays the determining role in achieving a desirable nanotherapeutic outcome. In addition, the change of reaction condition (e.g., elevating local temperature) or rational design of synergistic therapy (e.g., PDT/CDT, PTT/CDT, and chemotherapy/CDT) can also augment the therapeutic efficacy. The second consideration is the degradability and biocompatibility of the designed and fabricated Cu‐involved nanocatalysts. The balance of released Cu species for catalyzing Fenton‐like reaction and possibly induced toxicity should be considered. In addition, the following researches should concentrate more on the in vivo characterizations of catalytic procedures and mechanisms, by which the catalytic performances of these Cu‐involved nanocatalysts would be further optimized and improved.

## 4 Cu‐Involved Nanocarriers for Drug Delivery

Based on the advance of nanosynthetic chemistry, Cu‐involved nanosystems can be engineered with versatile nanostructures and compositions. For instance, the Cu‐based nanoparticles have been either integrated into the matrix of organic nanosystems or coated with a mesoporous silica shell, both of which provide the reservoirs for the encapsulation and delivery of therapeutic drug molecules toward chemotherapy. Their typical photothermal‐conversion effect assists the drug delivery by photothermal‐controlled on‐demand drug release and synergistic PTT/chemotherapy. In addition, the Cu‐involved component can be hybridized into the frameworks of nanocarriers for activating Cu‐participated chemotherapy. These inorganic Cu‐involved nanocarriers have also been designed and constructed with nanoporous and/or hollow nanostructures for efficient loading of guest drug molecules.

**Figure 12:**
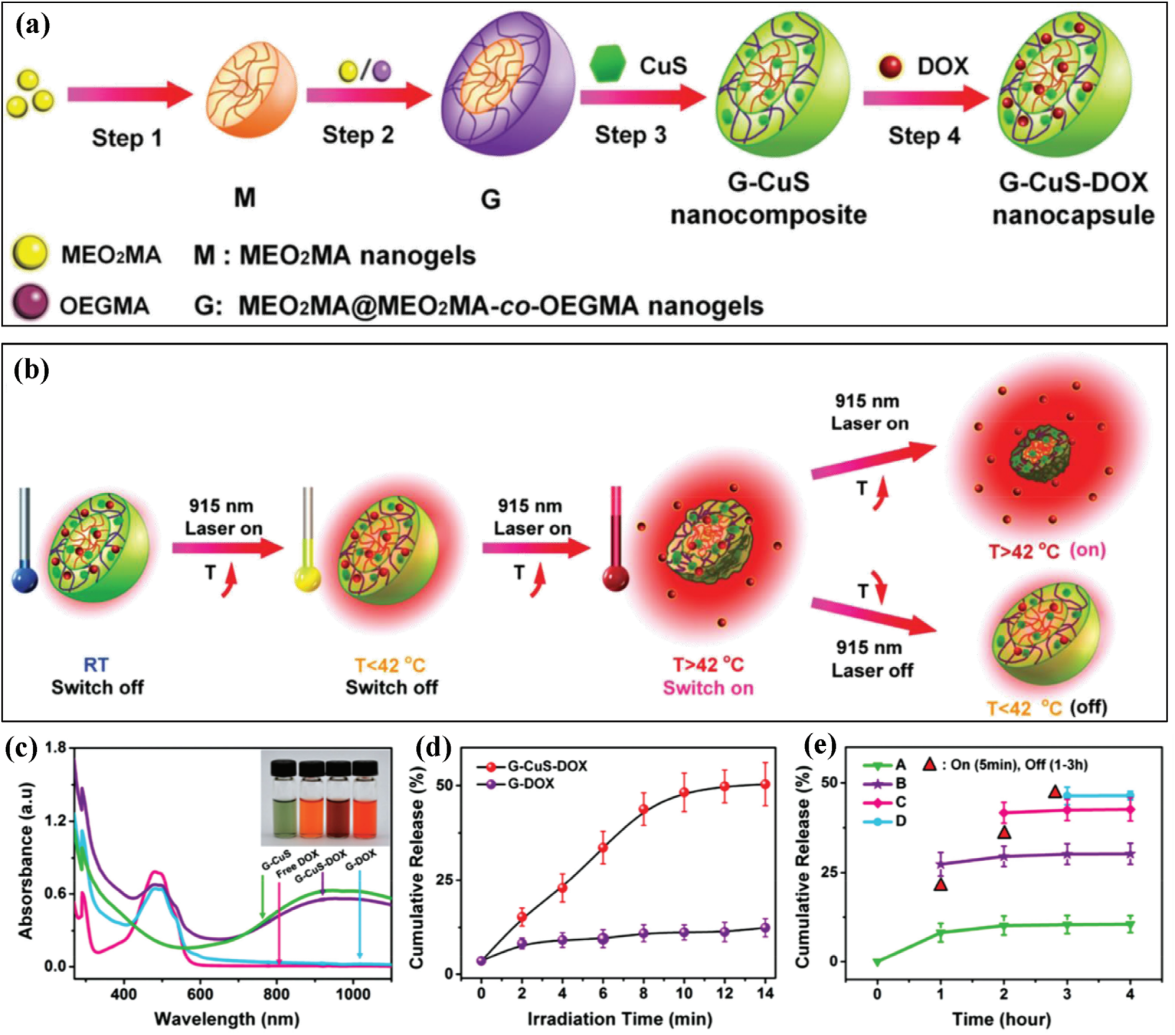
a,b) Schematic illustration on the construction of MEO_2_MA@MEO_2_MA‐*co*‐OEGMA‐CuS‐DOX nanocapsules (abbreviated as G‐CuS‐DOX). c) UV–vis absorbance spectra of different agents with the inset photographic image, including G‐CuS, free DOX, G‐GuS‐DOX, and G‐DOX. d) The DOX‐releasing profiles from either G‐CuS‐DOX or G‐DOX under the NIR irradiation (915 nm laser, 2.0 W cm^−2^). e) The releasing percentage of DOX with different irradiation–nonirradiation cycle numbers under 915 nm laser activation (1.6 W cm^−2^, one cycle: 5 min ON/5 min OFF, from A to D: 0, 1, 2, and 3 times). Reproduced with permission.^[^ ^117^ ^]^ Copyright 2016, Wiley‐VCH.

The photoresponsive property of Cu‐involved drug‐delivery nanosystems can be employed for manipulating the drug‐releasing behaviors. For instance, the MEO_2_MA@MEO_2_MA‐*co*‐OEGMA‐CuS‐DOX nanocomposite (designated as G‐CuS‐DOX) was stepwise constructed for photothermal‐controlled DOX release and chemotherapy.^[^ ^117^ ^]^ The thermosensitive MEO_2_MA@MEO_2_MA‐*co*‐OEGMA (abbreviated as G) nanogels was chosen because of their adequate low critical solution temperature (LCST) of 42 °C, which was used for the loading of both DOX and CuS nanoparticles (**Figure** **12**a). Based on the external NIR irradiation, the photothermal effect of CuS nanoparticles achieved the off/of switching of nanogels for controllable DOX release based on the LCST of 42 °C (Figure 12b). The high drug‐loading capability was demonstrated by UV–vis absorbance spectra (Figure 12c), and the photothermal‐triggered DOX release was evidenced by the DOX‐releasing profiles (Figure 12d) and cumulative DOX‐releasing amount under different NIR‐triggering modes (Figure 12e), where the intermittent NIR irradiation induced significantly higher DOX release from the nanocarrier. The synergistic photothermal ablation and photocontrollable chemotherapy realized high tumor‐suppression efficacy as demonstrated on tumor‐bearing mice model. Similarly, Cu_1.75_S nanoparticles were coated by a pH/thermo‐responsive polymer with the high DOX‐loading amount of 40 wt%, which exhibited both pH and photothermal sensitivities for the adaptable and sustained release of the loaded DOX, inducing synergistic antitumor effect on suppressing the growth of skin B16 melanoma tumor xenograft.^[^ ^118^ ^]^

**Figure 13:**
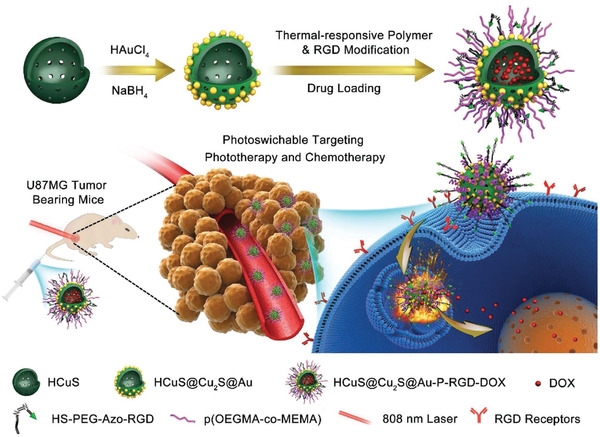
Schematic illustration on the construction of HCuS@Cu_2_S@Au‐P‐RGD nanoparticles, and their specific functionality of photoswitchable targeting, drug delivery for chemotherapy, and NIR‐induced photothermal ablation on U87MG tumor‐bearing mice. Reproduced with permission.^[^ ^119^ ^]^ Copyright 2017, Wiley‐VCH.

Hollow‐structured CuS@Cu_2_S@Au nanoshell/satellite composite nanoparticles with surface photoswitchable RGD targeting modification (abbreviated as HCuS@Cu_2_S@Au‐P‐RGD) were constructed as the drug‐delivery nanocarriers for tumor therapy (**Figure** **13**).^[^ ^119^ ^]^ Au nanoparticles were decorated onto the surface based on the consideration that the Au component could change the electron transitions in the semiconductor moieties for enhancing the photothermal‐conversion performance. Especially, the large hollow interior provided the reservoirs for the encapsulation of anticancer drug DOX to realize chemotherapy. The photoswitchable targeting transportation to U87MG tumor was achieved by surface‐modified thermal‐isomerization RGD molecules. The targeted chemotherapy combined with the enhanced photothermal ablation induced a high tumor‐inhibitory effect on U87MG tumor‐bearing mice by intravenous administration.

**Figure 14:**
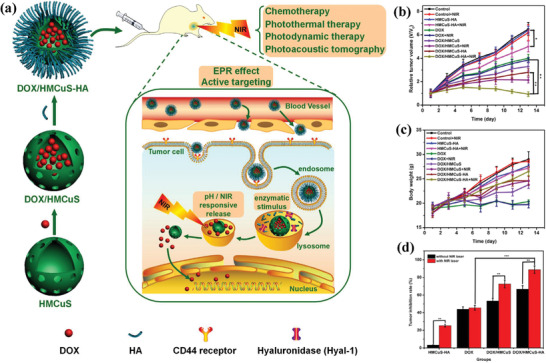
a) Schematic illustration on the construction of multifunctional DOX/HMCuS‐HA targeted drug‐delivery nanosystem for PA imaging‐guided synergistic chemotherapy and photothermal hyperthermia. b) Tumor volume‐changing curves of tumor‐bearing mice after varied treatments, and c) the corresponding body‐weight changes. d) Tumor‐inhibiting rates in different therapeutic groups. Reproduced with permission.^[^ ^120^ ^]^ Copyright 2017, Elsevier.

Based on the targeting capability of hyaluronic acid (HA), the targeted drug delivery nanosystems were constructed by engineering hollow mesoporous copper sulfide nanoparticles (HMCuS), where the large hollow interior provided the reservoirs for chemotherapeutic drug DOX (**Figure** **14**a).^[^ ^120^ ^]^ The further surface HA modification endowed the HMCuS nanocarriers with targeting functionality into MCF‐7 cancer cells by CD44 receptor‐mediated endocytosis pathway. The synergistic chemotherapy and NIR‐induced photonic ablation substantially suppressed the tumor growth (Figure 14b) with improved body‐weight increase (Figure 14c) as compared to free DOX treatment. The tumor‐inhibition rate in the synergistic therapeutic group reached 88.9%, much higher than HMCuS‐HA/NIR group (25.4%) and DOX only group (43.9%), signifying the high synergy. In addition, hollow CuS nanoparticles were fabricated employing Cu_2_O nanoparticles as the template for the delivery of hydrophobic anticancer drug camptothecin (CPT), in accompany with further 980 nm laser‐activated photothermal ablation for in vivo synergistic cancer therapy on combating H22 tumor.^[^ ^121^ ^]^

Based on the extensively explored mesoporous silica nanoparticles (MSNs) for drug delivery,^[^ ^122^, ^123^, ^124^, ^125^ ^]^ Cu‐based nanoparticles have been integrated with mesoporous silica nanoparticles by four strategies for endowing them with drug‐loading capability, including coating a mesoporous silica layer onto the surface of them, they are grown into mesopores or onto the surface of MSNs and hybridization of Cu‐based components into MSNs’ framework. For instance, the surface of Cu_9_S_5_ nanoparticles was coated by a mesoporous silica layer with further PEGylation for the loading of anticancer drug DOX, which was realized by the adoption of cetyltrimethylammonium bromide (CTAB, as the pore‐making agent) for transforming hydrophobic Cu_9_S_5_ nanocrystals into aqueous solution followed by the hydrolysis/condensation of tetraethylorthosilicate (TEOS, as the silica source).^[^ ^126^ ^]^

CuS nanoparticles were in situ grown into the mesopores of periodic mesoporous organosilica (designated as CuS@PMO) for achieving dual functionalities.^[^ ^127^ ^]^ The well‐defined mesopores in PMOs enabled the high loading amount (470 mg g^−1^) of chemotherapeutic drug DOX, and the CuS component elevated the local temperature to trigger the DOX release from the mesopores. The CuS‐enabled mild hyperthermia effect also enhanced the intracellular uptake of CuS@PMO for strengthening the synergistic therapeutic efficacy of DOX chemotherapy and CuS photothermal ablation, achieving high tumor‐suppressing consequence on U87MG tumor‐bearing mice. In addition, CuS nanoparticles were decorated onto the surface of MSNs with further PEGylation for the encapsulation and delivery of chemotherapeutic drug DOX by mesopores. The photothermal‐triggered DOX release and chemotherapy were enabled by the decorated CuS nanoparticles with synergy for the photothermal ablation of hepatocellular carcinoma.^[^ ^128^ ^]^

**Figure 15:**
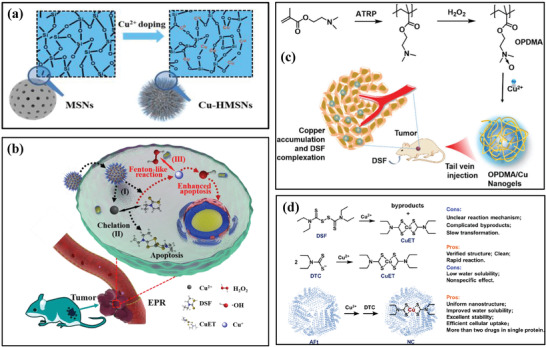
a) Schematic illustration of framework composition of mesoporous silica nanoparticles and Cu‐incorporated hollow mesoporous silica nanoparticles. b) The scheme of the fabricated DSF@PEG/Cu‐HMSNs nanoparticles for synergistic in situ produced CuET‐based chemotherapy and ROS‐based oxidative therapy. Reproduced with permission.^[^ ^129^ ^]^ Copyright 2019, American Chemical Society. c) Schematic clarification of the construction of OPDMA/Cu nanogel for delivering Cu ions into the tumor for further chelating with DSF toward tumor chemotherapy. Reproduced with permission.^[^ ^130^ ^]^ Copyright 2020, Royal Society of Chemistry. d) The scheme showing the reaction between DSF and copper to form CuET, DTC and copper to form CuET, and in situ encapsulation of CuET into AFt nanocarriers. Reproduced with permission.^[^ ^131^ ^]^ Copyright 2020, American Chemical Society.

Disulfiram (DSF) as an FDA‐approved drug for the treatment of alcohol dependence, is demonstrated to be effective in cancer therapy, which strongly depends on the presence of copper ions.^[^ ^132^ ^]^ In detail, DSF can chelate Cu ions to form dithiocarbamate (DTC)‐copper complexes (CuETs). The postgenerated CuETs are featured with a profoundly improved antitumor effect. The traditional strategy for Cu ions supplying is the intraperitoneal administration of free ions, which however would cause the potential toxicity. Based on this fact, we recently directly incorporated Cu ions into the framework of hollow mesoporous silica nanoparticles (Cu‐HMSNs; **Figure** **15**a) for the construction of Cu‐involved nanocarriers with both DSF‐loading capacity and Cu‐supplying property.^[^ ^129^ ^]^ The mildly acidic condition of the tumor microenvironment readily broke up the framework of Cu‐HMSNs, enabling the release of loaded DSF and Cu ions. The in situ chelation of Cu ions and DSF produced toxic CuETs for inducing chemotherapeutic effect, and the Cu species acted as Fenton species for converting tumor‐overexpressed H_2_O_2_ into hydroxyl radicals, inducing synergistic effect on chemotherapy (Figure 15b). A tertiary amine‐oxide‐based zwitterionic polymer (poly[2‐(*N*‐oxide‐*N*,*N*‐dimethyl‐amino)ethyl methacrylate], OPDMA) with Cu‐complexing capability was synthesized for delivering copper ions into tumor (Figure 15c). After oral administration of DSF, the tumor‐delivered Cu ions chelated with the tumor‐accumulated DSF for generating toxic CuET toward anticancer chemotherapy with high tumor‐suppression rate. Based on the Cu‐bound proteins, the horse spleen apoferritin (AFt) was developed as the nanocarriers to in situ noncovalently immobilize CuET based on the specific binding affinity of Aft toward Cu ions (Figure 15d).^[^ ^131^ ^]^ AFt as the CuET nanocarriers could enhance the bioavailability of CuET with low water solubility, which was further employed for co‐loading of DOX to achieve synergistic chemotherapeutic efficiency.

**Figure 16:**
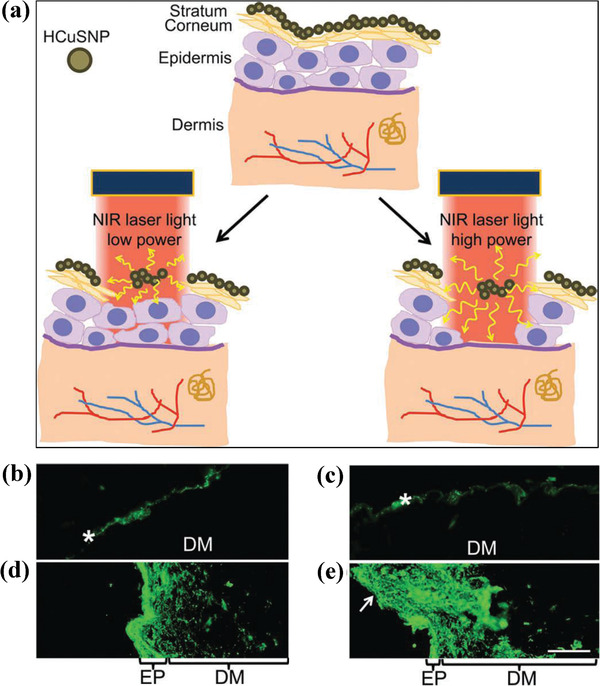
a) Schematic illustration of HCuSNP‐enabled photothermal ablation of skin tissue by external NIR irradiation. Fluorescent images of dextran‐FITC treated skin section of anesthetic nude mice after the pretreatment with b) HCuSNP gel, c) blank gel + laser (2.6 W cm^−2^), d) HCuSNP gel + laser (1.3 W cm^−2^), and e) HCuSNP gel + plus (2.6 W cm^−2^). EP: epidermis; DM: dermis. Reproduced with permission.^[^ ^133^ ^]^ Copyright 2012, Wiley‐VCH.

The photothermal ablation capability of hollow copper sulfide nanoparticles (HCuSNPs) was employed for developing transdermal delivery of therapeutic agents, which were suspended in Carbomer 940 hydrogel.^[^ ^133^ ^]^ The HCuSNP‐enabled photothermal energy conversion ablated the stratum corneum by adaptable NIR power density (**Figure** **16**a), which effectively enhanced the drug permeability. Dextran‐FITC was used as the model drug molecules to evaluate the skin penetration of hydrophilic molecules after the photothermal ablation of tissue. It was demonstrated that the strong green fluorescence was observed throughout the epidermis and even penetrating into the dermis layer after the treatment with NIR‐irradiated HCuSNP gel, and the use of higher NIR power density enabled deeper penetration of dextran‐FITC (Figure 16b–e). The same phenomenon was observed by the transdermal delivery of human growth hormone (hGH) with improved elevation in the transdermal flux of hGH as compared to traditional transdermal enhancement patches. This drug‐delivery biomaterial platform provides a sustained and adaptable strategy for the transdermal delivery of drug molecules with varied molecular sizes.

The dominant design principle of Cu‐involved drug‐delivery nanosystem is the creation of space or reservoirs for accommodating therapeutic drug molecules. Two strategies have been generally adopted, including engineering Cu‐involved nanoparticles with hollow interiors or mesoporous nanostructures, and integrating Cu‐involved nanoparticles into other nanoagents or bulk matrix with the drug‐loading capacity. It is noted that the typical photothermal‐conversion performance has been typically employed to achieve thermal‐sensitive drug release and synergistic photothermal hyperthermia/chemotherapy. Because of the intrinsic multifunctionality of Cu composition, these drug‐delivery systems exhibit superior therapeutic performance as compared to traditional mesoporous silica or some organic nanosystems such as liposomes, but the critical issues of low biodegradation rate and toxicity of released Cu ions of some inorganic Cu‐based nanosystems should be further addressed.

## 5 Cu‐Involved Nanoagents for Antibacterial Applications

Bacterial infections are one of the serious diseases threatening human health such as the complication after implantation surgery,^[^ ^134^, ^135^, ^136^, ^137^, ^138^ ^]^ but the traditional antibiotics usually induce the antibiotic resistance of bacteria, which severely hinders the treatment efficacy of bacterial infections.^[^ ^139^, ^140^, ^141^ ^]^ Metal‐based (e.g., Au, Ag, and Cu) nanoparticles as the alternative bactericides of traditional antibiotics have aroused ever‐increasing attention because of their high stability, distinctive antibacterial nature and specific performance against multidrug‐resistant bacteria.^[^ ^142^, ^143^, ^144^, ^145^, ^146^, ^147^, ^148^ ^]^ Especially, Cu‐involved components are the desirable antimicrobial agents on combating versatile bacteria with some underlying mechanisms including the generation of ROS, released metal‐ion toxicity and the potential mechanical destruction of bacterial cell walls/membranes.^[^ ^149^, ^150^, ^151^, ^152^, ^153^, ^154^, ^155^, ^156^, ^157^ ^]^

Copper nanoparticles were revealed to be effective in antibacteria against *Micrococcus luteus*, *Staphylococcus aureus*, *Escherichia coli*, *Klebsiella pneumoniae*, and *Pseudomonas aeruginosa*.^[^ ^158^ ^]^ The further decoration of copper nanoparticles into inexpensive mineral vermiculite was featured with antibacterial activity against *S. aureus*, showing the promising antibacterial use of copper‐involved hybrid materials.^[^ ^159^ ^]^ Copper nanoparticles were also coated onto cellulose films with desirable antibacterial bioactivity against *S. aureus* and *E. coli*.^[^ ^160^ ^]^ Copper iodide (CuI) nanoparticles were exemplified to produce ROS in both gram‐negative and gram‐positive bacteria for inducing antibacterial activity by DNA and membrane damage.^[^ ^161^ ^]^ Copper oxide (CuO) nanocrystals exhibited the minimum inhibitory concentration of 2.5 µg mL^−1^ on inhibiting *E. coli* organism by damaging the cell envelope irreversibly.^[^ ^162^ ^]^ The CuO nanoparticles were also featured with a shape‐dependent antibacterial activity where the plate‐like CuO featured higher antibacterial performance as compared to grain or needle‐shaped CuO nanomaterials.^[^ ^163^ ^]^ Detailed mechanism investigation revealed that the formation of Cu_2_O‐originated copper(I)‐peptide complex and CuO‐induced free radicals induced the antibacterial effect against *E. coli*.^[^ ^164^ ^]^ Especially, the synergistic photothermal and photodynamic effect of CuS nanoparticles induced high antibacterial efficacy against *S. aureus* (99.80%) and *E. coli* (99.94%).^[^ ^165^ ^]^ In addition, the inclusion of Cu^2+^ into calcium silicate coatings on titanium metal endowed the biocoating with antibacterial performance against *E. coli* and *S. aureus*.^[^ ^166^ ^]^ The fabricated copper‐containing mesoporous bioactive glasses (MBGs) also exhibited a desirable antibacterial effect on killing *E. coli*, *S. aureus*, and *Staphylococcus epidermidis*, and further disrupting the *S. epidermidis*‐induced biofilm dispersion.^[^ ^167^ ^]^

**Figure 17:**
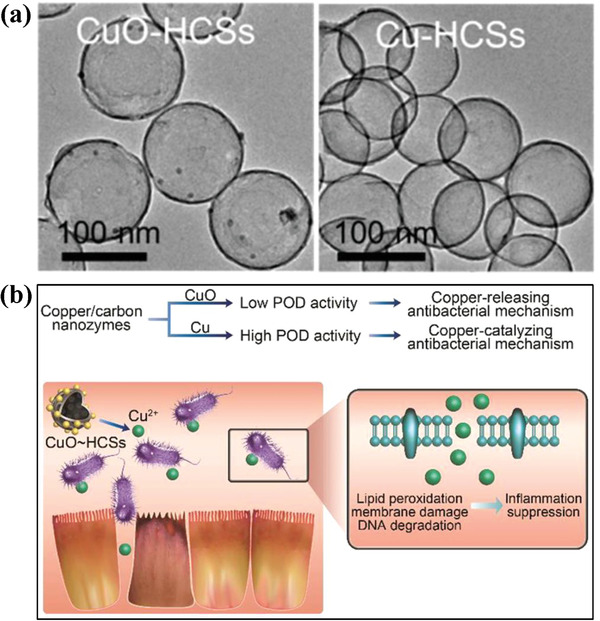
a) TEM images of CuO‐modified copper/carbon nanoenzymes (CuO‐HCSs) and Cu‐modified copper/carbon nanoenzymes (Cu‐HCSs). b) Schematic illustration of the underlying antibacterial mechanism of either CuO‐HCSs or Cu‐HCSs nanoenzymes. Reproduced with permission.^[^ ^168^ ^]^ Copyright 2019, American Chemical Society.

Based on the fact that the integration of metal or metal oxides could improve the enzyme‐mimicking activity of carbon nanoenzymes, two types of copper/carbon hybrid nanospheres, i.e., CuO‐modified copper/carbon nanoenzymes (CuO‐HCSs) and Cu‐modified copper/carbon nanoenzymes (Cu‐HCSs; **Figure** **17**a), were constructed for antibacterial applications, which exhibited Cu state‐dependent enzyme‐mimicking activities, including peroxidase, catalase and superoxide dismutase.^[^ ^168^ ^]^ Especially, the CuO‐HCSs and Cu‐HCSs possessed different underlying antibacterial mechanisms. For CuO‐HCSs, the released Cu^2+^ induced the gram‐negative bacterial toxicity by bacterial membrane damage, lipid peroxidation and DNA degradation. Comparatively, the constructed Cu‐HCSs nanoenzymes triggered the peroxidase‐mimicking catalytic reactions to produce ROS, which caused the oxidative damage of both gram‐positive and gram‐negative bacteria (Figure 17b). The high antibacterial effect was also demonstrated in vivo on bacteria‐infected animal models. This study provides a distinctive strategy on the rational design of Cu‐based nanoenzymes for antibacterial use. In addition, the construction of AgCu nanoalloys displayed enhanced antibacterial bioactivity against *E. coli* as compared to equivalent Ag nanoparticles as the traditional inorganic antibacterial nanoagent.^[^ ^169^ ^]^

**Figure 18:**
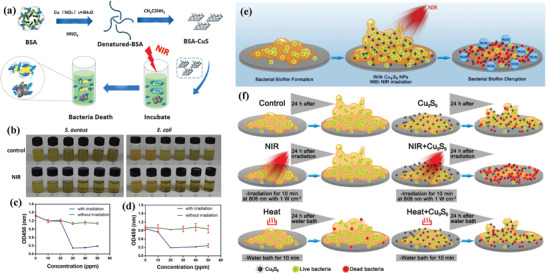
a) Schematic illustration on the construction of BSA‐CuS nanoparticles for NIR‐induced photothermal ablation on killing bacteria. b) Photographic images showing turbidity changes of *S. aureus* and *E. coli* after incubation with CuS nanoparticles at elevated concentrations (0, 10, 20, 30, 40, and 50 ppm) followed with or without NIR irradiation (1.59 W cm^−2^, 45 min), and the corresponding growth curves of c) *S. aureus* and d) *E. coli* at varied concentrations in the presence or absence of NIR irradiation. Reproduced with permission.^[^ ^131^ ^]^ Copyright 2017, American Chemical Society. e) The scheme of destroying *S. aureus* biofilm by NIR‐activated Cu_9_S_8_ nanoparticles based on both photothermal and photodynamic effects, and f) the detailed changes of bacterial biofilm after three treatments, including Cu_9_S_8_, NIR‐activated Cu_9_S_8_, and Cu_9_S_8_ plus heat treatment. Reproduced with permission.^[^ ^171^ ^]^ Copyright 2019, American Chemical Society.

CuS nanoparticles are featured with high biocompatibility but without antibacterial activity. Their intrinsic photothermal effect is effective for exerting the antibacterial function. For instance, bovine serum albumin (BSA)‐templated CuS nanoparticles exhibited satisfactory antibacterial bioactivity on killing two most common infectious bacteria *S. aureus* and *E. coli* with a 980 nm laser irradiation at the power density of 1.59 W cm^−2^ (**Figure** **18**a).^[^ ^170^ ^]^ Such an antibacterial effect was also concentration‐dependent (Figure 18b–d) where the turbid bacteria suspend turned into a clear solution because of the lost biological activity of bacteria after NIR irradiation. Based on the varied architecture of bacterial walls, the CuS‐induced antibacterial mechanism on *S. aureus* and *E. coli* by photothermal ablation was different. In addition, the biofilms are commonly formed by bacterial attachment and further generation of extracellular polymers, which can hinder the penetration of antibiotics for killing bacteria. Both photothermal and photodynamic effects of Cu_9_S_8_ nanoparticles were employed for destructing bacterial biofilm (*S. aureus*) on the Ti plates, where the photodynamic ROS production and photothermal hyperthermia induced synergistic antibacterial consequences (Figure 18e). It was much more efficient as compared to the single photothermal effect of Cu_9_S_8_ nanoparticles (Figure 18f).^[^ ^171^ ^]^ Albumin‐stabilized CuS nanoagents exhibited photothermal‐augmented antibacterial activity on killing multidrug‐resistant bacteria upon NIR irradiation, including methicillin‐resistant *S. aureus* and extended‐spectrum *β*‐lactamase *E. coli*. In addition, the released Cu^2+^ from CuS nanoparticles accelerated the healing of chronic nonhealing wounds with multidrug‐resistant bacterial infection by facilitating the migration of fibroblast cells and angiogenesis of endothelial cells.^[^ ^172^ ^]^

## 6 Cu‐Involved Nanoagents for Tissue Engineering

It has been extensively revealed and demonstrated that Cu ions are effective for tissue regeneration especially for skin‐tissue regeneration because of their bioactivity on enhancing angiogenesis, cell migration, and collagen deposition. Therefore, Cu‐involved nanoagents or biomaterial platforms have been elaborately constructed for tissue‐engineering applications.^[^ ^173^ ^]^ For instance, based on the specific capability of Cu^2+^ ions for improving angiogenesis, Cu‐containing bioactive glass (BG)/eggshell membrane (ESM) nanocomposite film was constructed by pulsed laser deposition technique for wound healing of skin tissue.^[^ ^174^ ^]^ The fabricated Cu‐involved BG/ESM nanocomposite films exhibited high wound‐healing bioactivity by enhancing the angiogenesis‐correlated gene expression and protein secretion of VEGF and HIF‐1*α*, in accompany with the desirable antibacterial activity.

Copper‐containing mesoporous bioactive glass (Cu‐MBG) was combined with nanofibrillated cellulose (NFC) for the controllable release of Cu, facilitating the wound healing by inducing a profound angiogenic effect, in accompany with the antibacterial bioactivity against gram‐negative *E. coli*.^[^ ^175^ ^]^ The Cu deposition into titanium implants not only inhibited the growth of bacteria (*S. aureus*) but also stimulated the proliferation and enhanced the osteogenic differentiation of human bone marrow‐derived mesenchymal stem cells (MSCs).^[^ ^176^ ^]^ The balance of Cu concentration was the determinant because the high‐concentration Cu ions induced the antibacterial infection but the low‐concentration Cu ions promoted the bone regeneration.

**Figure 19:**
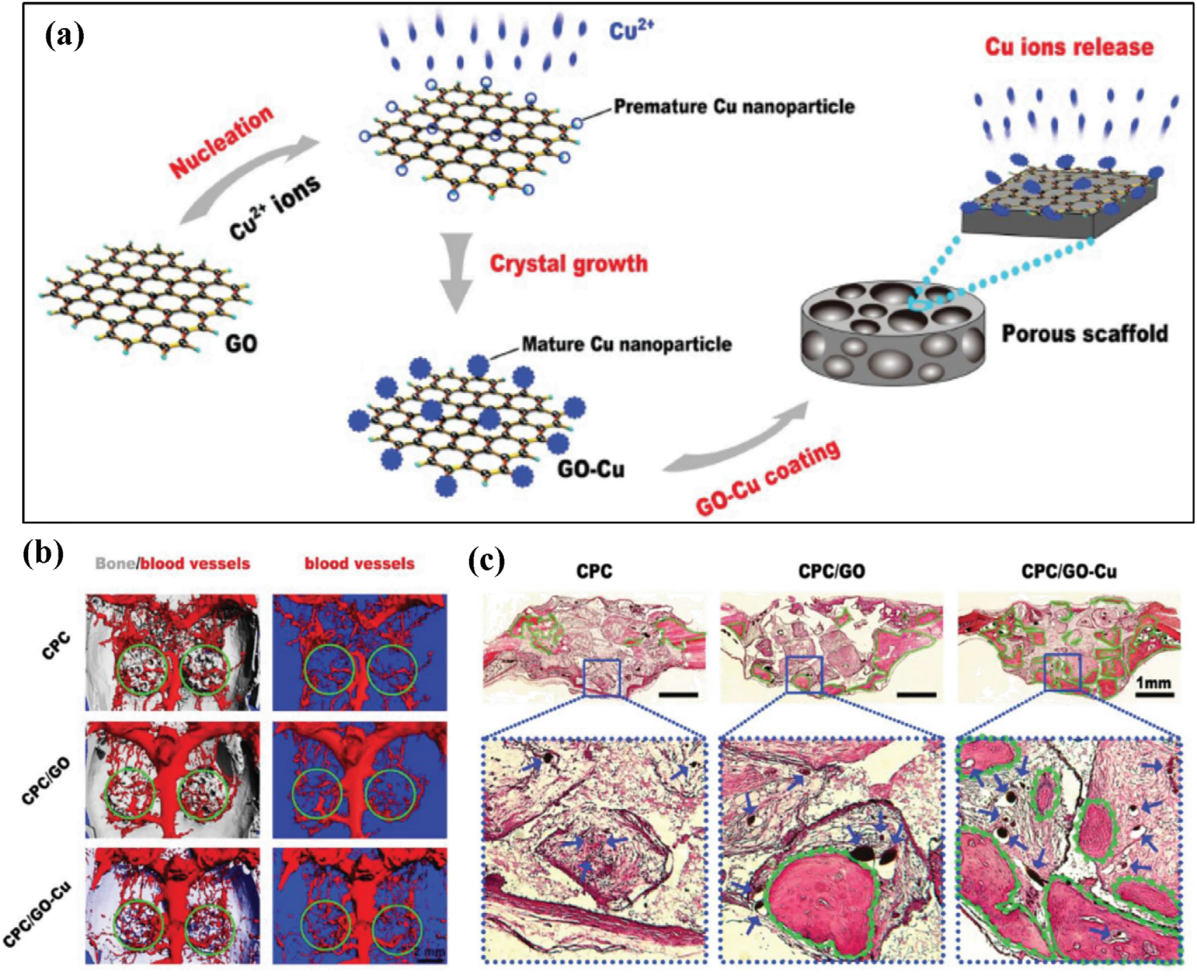
a) Schematic illustration on the construction of GO–Cu‐coated CPC composite scaffolds. b) Micro‐CT images of the blood vessels of rat cranial bone defects as perfused with Microfil after varied treatments, and c) the corresponding histological analysis of newly formed bone tissues. Reproduced with permission.^[^ ^177^ ^]^ Copyright 2016, Wiley‐VCH.

Graphene oxide–copper (GO–Cu) nanocomposites were coated onto the surface of calcium phosphate cement (CPC) scaffolds (abbreviated as CPC/GO–Cu) for yielding vascularized bone regeneration (**Figure** **19**a), which was based on the fact that graphene was capable of promoting the osteogenic differentiation of bone marrow stem cells (BMSCs) and Cu‐involved nanoparticles could maintain the bone volume and expedite the bone‐healing rate.^[^ ^177^ ^]^ The integrated GO–Cu nanocomponents only promoted the adhesion and osteogenic differentiation of rate BMSCs in vitro by augmenting the expression of VEGF and BMP‐2, but also accelerated the in vivo angiogenesis (Figure 19b) and osteogenesis (Figure 19c). The doping of copper into MSNs with uniform spherical topology and the well‐defined mesoporous structure was exemplified to be phagocytized by immune cells for modulating the immune microenvironment, which could induce osteogenic/angiogenic factors and inhibit osteoclastogenic factors. This paradigm reveals the possibility of Cu‐doped biomaterials as an immunomodulatory agent for inducing osteogenesis and subsequent bone regeneration.^[^ ^178^ ^]^

**Figure 20:**
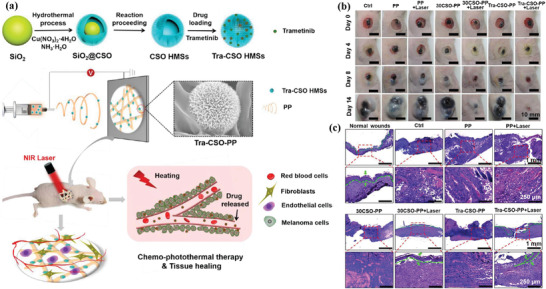
a) The fabrication procedure of Tra‐CSO‐PP scaffolds (Tra: Trametinib drug; CSO: copper silicate hollow microspheres (CSO HMSs); PP: poly(*ε*‐caprolactone/poly(d,l‐lactic acid) fibrous scaffolds) and their specific functionality of synergistic chemo‐photothermal therapy of melanoma skin cancer and promotion of skin‐tissue regeneration. b) The photographic images of the tumors and skin wounds during the therapeutic period in different treatment groups, and c) corresponding H&E staining images of skin wounds after the treatment for 14 days in varied groups. Reproduced with permission.^[^ ^179^ ^]^ Copyright 2018, American Chemical Society.

The melanoma treatment typically requires the simultaneous killing of cancer cells by different therapeutic modalities and skin‐tissue regeneration for repairing the damaged tissue. The therapeutic and regeneration bifunctionalities of Cu‐based nanoagents provide the unique theragenerative biomaterial platform for melanoma treatment. As a typical paradigm, copper silicate hollow microspheres (CSO) were integrated into bioactive scaffolds for synergistic chemotherapy and photothermal ablation of melanoma, and skin‐tissue regeneration (**Figure** **20**a),^[^ ^179^ ^]^ which originated from the promoted proliferation and attachment of normal skin cells, and stimulated revascularization and re‐epithelialization. The photothermal tumor therapy did not influence the wound‐healing procedure (Figure 20b), and the epidermis in the group of CSO‐integrated scaffolds achieved the thickest formation with normal architecture compared to other control groups (Figure 20c). Similarly, Cu_2_S nanoparticles were embedded into a biopolymer fiber for inhibiting skin‐tumor growth and skin‐regeneration.^[^ ^180^ ^]^ Cu‐based metal–organic framework (Cu‐MOF; HKUST‐1) nanoparticles were featured with sustained Cu^2+^‐releasing property with the modification of folic acid. They could promote the angiogenesis and collagen deposition for promoting the healing of chronic nonhealing wounds as demonstrated in diabetic mice with splinted excisional dermal wounds.^[^ ^181^ ^]^ These Cu‐MOF nanoparticles were also embedded into citrate‐based hydrogel with antioxidant thermo‐responsive property for mitigating the toxicity of Cu ions and strengthening the wound‐healing process.^[^ ^43^ ^]^

**Figure 21:**
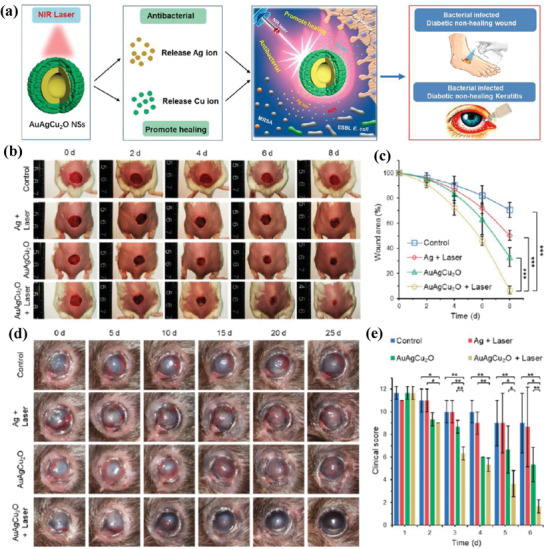
a) Schematic illustration of the underlying functionality of AuAgCu_2_O nanoshells for killing antibacterial‐resistant bacteria and accelerating wound healing. The photothermal effect and released Ag ions induced synergistic antibiotic and photothermal effect for killing bacteria, and the sustained release of Cu ions promoted the recovery of nonhealing sound and keratitis. b) Photographic images of MRSA‐infected full‐thickness dorsal cutaneous incisions after varied treatments as indicated in the image for prolonged durations on BALB/c mice, and c) corresponding wound‐area closure ratios. d) Photographic images of MRSA‐infected chronic nonhealing keratitis after different treatments on diabetic mice for a prolonged time, and e) the corresponding clinical score evaluations including opacity area, surface regularity, and opacity density. Reproduced with permission.^[^ ^182^ ^]^ Copyright 2020, American Chemical Society.

AuAgCu_2_O hollow nanoshell was constructed with a hollow AuAg core and Cu_2_O shell for combating drug‐resistant bacterial infection and accelerating the recovery of cutaneous chronic wound and nonhealing keratitis (**Figure** **21**a).^[^ ^182^ ^]^ The photothermal effect of AuAgCu_2_O hollow nanoshell and sustained Ag ions release induced the synergistic antibacterial effect on killing multidrug‐resistant bacteria, such as the extended‐spectrum *β*‐lactamase *E. coli* (ESBL *E. coli*) and methicillin‐resistant *S. aureus* (MRSA). Importantly, these AuAgCu_2_O hollow nanoshell expedited the wound‐healing effects by the sustained release of Cu ions from Cu_2_O component, as demonstrated in the treatment of MRSA‐infected cutaneous wound with improved recovery after the treatment with laser plus AuAgCu_2_O nanoshells (Figure 21b,c) and MRSA‐infected keratitis model in diabetic mice with promoted elimination of MSRA and recovery of the cornea (Figure 21d,e). This paradigm reveals that the Cu‐involved nanosystems could be engineered with concurrent antibacterial and tissue‐generating capabilities for treating chronic wound and nonhealing keratitis as caused by MRSA infection.

## 7 Cu‐Involved Nanoagents for Synergistic Nanotherapeutics

Because of the complexity of tumor such as the specific TME and tumor metastasis, it is the hurdle for completely eradicating the tumor simply by monotherapy. The development of some distinctive therapeutic modalities with multiple and synergistic therapeutic performance is highly prospectus for yielding strengthened therapeutic efficacy and outcome.^[^ ^183^, ^184^, ^185^, ^186^, ^187^, ^188^ ^]^ Therefore, the rational design of optimal portfolios is of high significance for achieving desirable synergistic therapeutic consequences. On this ground, some distinctive Cu‐involved multifunctional nanosystems have been constructed for producing multiple nanotherapeutics with synergy.^[^ ^189^ ^]^

**Figure 22:**
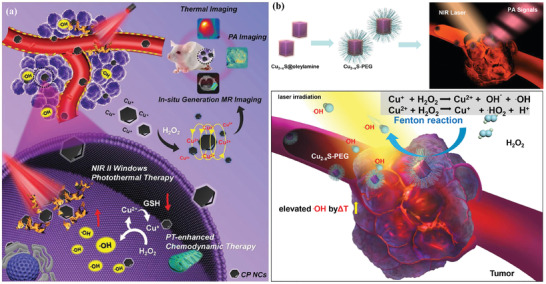
Schematic illustration of the underlying synergistic therapeutic mechanism of copper(I) phosphide nanocrystals (CP NCs) for multimodality imaging‐guided photothermal ablation and photothermal‐enhanced CDT of the deep‐seated tumor. Reproduced with permission.^[^ ^190^ ^]^ Copyright 2019, Wiley‐VCH. b) The scheme of the synthesis of PEGylated Cu_2−_ *_x_*S nanodots for synergistic and photothermal‐enhanced Fenton catalysis and oxidative tumor therapy under the guidance of PA imaging. Reproduced with permission.^[^ ^191^ ^]^ Copyright 2019, Elsevier.

Copper(I) phosphide nanocrystals (CP NCs) with intrinsic photothermal‐conversion performance were constructed for photothermal ablation and photothermal‐enhanced Fenton‐like reaction‐induced oxidative cancer therapy (**Figure** **22**a).^[^ ^190^ ^]^ Based on the reactivity of Cu component, the tumor‐overexpressed H_2_O_2_ was converted into hydroxyl radicals (•OH) for inducing oxidative tumor therapy. The postgenerated Cu(II) was reduced into Cu(I) by excess GSH in tumor conditions for continuously supplying reactive catalytic centers. Based on the LSPRs, these CP NCs responded to external photonic activation at the second NIR biowindow for photothermal tumor ablation. Because of the temperature sensitivity, the elevated local temperature in tumor further accelerated the rate and efficacy of Fenton reaction, inducing synergistic tumor‐therapeutic consequence. In addition to the photothermal and photodynamic effect of Cu_2−_ *_x_*S nanoparticles, their catalytic performance on Fenton reaction was triggered under the mildly acidic condition of tumor microenvironment for producing hydroxyl radicals (Figure 22b), and the Fenton efficacy was also strengthened by the NIR‐II‐induced photothermal effect, resulting in synergistic photothermal ablation and catalytic nanotherapeutics against 4T1 tumors under the guidance of PA imaging.^[^ ^191^ ^]^ Similarly, hollow Cu_2_Se nanoenzymes also exhibited the NIR‐II‐activated photothermal ablation‐strengthened catalytic oxidative tumor therapy.^[^ ^192^ ^]^

**Figure 23:**
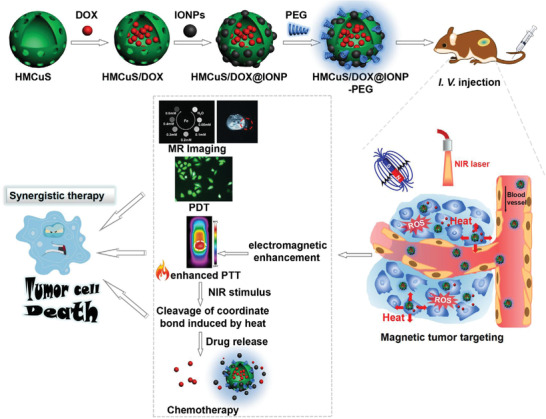
Schematic illustration on the construction of HMCnS/DOX@IONP‐PEG nanoparticles with the combined triple synergistic therapeutic modalities, including NIR‐induced photothermal ablation, photodynamic therapy, and controllable chemotherapy under the guidance and monitoring of MR imaging. Reproduced with permission.^[^ ^193^ ^]^ Copyright 2017, Elsevier.

Based on the intrinsic photothermal effect, the nanostructure design of Cu‐involved nanosystems can be further developed for drug delivery, yielding the synergistic photothermal hyperthermia and chemotherapy.^[^ ^194^, ^195^, ^196^, ^197^, ^198^ ^]^ To achieve higher synergistic therapeutic efficacy, the photothermal and photodynamic effects of hollow mesoporous CuS nanoparticles (designated as HMCuS NPs) were fabricated as the nanocarriers for chemotherapeutic drug DOX with the capped superparamagnetic iron oxide nanoparticles (abbreviated as HMCuS/DOX@IONP‐PEG; **Figure** **23**).^[^ ^193^ ^]^ Superparamagnetic nanoparticles enhanced the SPR effect for strengthening the PTT efficacy, acted as the caps for controllable DOX release by photothermal temperature elevation‐induced weakening of the coordination interaction between HMCuS NPs and IONPs, and enabled magnetically targeted transport of composite nanoparticles within the blood vessels. The triple synergistic photothermal ablation, PDT, and chemotherapy achieved the highest tumor‐inhibition outcome as compared to either single therapy or dual therapies. CuS nanoparticles were also anchored onto the surface of hollow MSNs for the delivery of DOX toward chemotherapy with synergistic photothermal ablation.^[^ ^199^ ^]^ Small‐sized CuS@mSiO_2_ core/shell nanoparticles exhibited similar functions for simultaneous anticancer drug delivery and photothermal hyperthermia of cancer cells with photothermal‐controlled drug‐releasing behavior.^[^ ^200^ ^]^ In addition, CuS was integrated with block copolymer‐based micelles, which were featured with an upper critical solution temperature of around 38 °C.^[^ ^201^ ^]^ The NIR‐induced photothermal effect of CuS component triggered the accelerated drug release from the nanocarrier by the temperature change‐enabled hydrophobic‐to‐hydrophilic transition. The synergistic effect of chemotherapy and PTT was demonstrated in a 3D multicellular tumor spheroid model. The design of coordinated polymer nanoparticles with the encapsulation of Cu(II) diethyldithiocarbamate (Cu(DDC)_2_) exhibited synergistic therapeutic outcome of chemotherapy and CDT, where Cu(DDC)_2_ exerted the chemotherapeutic function and the additional bioavailable Cu(II) out of Cu(DDC)_2_ catalyzed the production of hydroxyl radicals in oxidative tumor therapy.^[^ ^202^ ^]^

**Figure 24:**
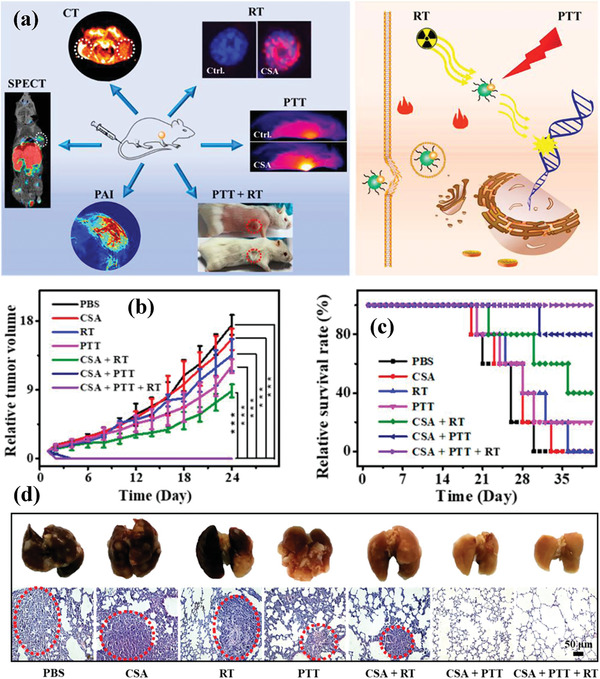
a) Schematic illustration of triple diagnostic imaging (PA/SPECT/CT)‐guided synergistic PTT and RT based on heterogeneous CSA nanoagents, and the underlying synergistic mechanism of PTT and RT. b) Tumor‐volume changes after varied treatments and c) corresponding survival rates. d) Photographic images of the lungs of tumor‐bearing mice at the end of diverse treatments, and corresponding H&E‐stained lung slices. Reproduced with permission.^[^ ^203^ ^]^ Copyright 2019, American Chemical Society.

The construction of Cu_2−_ *_x_*E–Au (E = S, Se) hetero‐nanostructure concurrently achieved augmented attenuation of X‐rays and enhanced attenuation coefficient via the multiple but synergistic interactions of X‐rays with different elements in composite nanosystems, which could also strengthen the photothermal‐conversion efficiency of Cu‐based semiconductors. On this ground, the heterogeneous copper selenide‐Au (CSA) nanoparticles with dumbbell morphology were synthesized as both radiosensitizer and photothermal nanoagent for synergistic RT and PTT against tumor (**Figure** **24**a), in accompany with triple‐diagnostic imaging capability by labeling with radioactive ^99m^Tc (SPECT, PA, and CT).^[^ ^203^ ^]^ Such a synergistic performance was much profound as compared to either Cu_2−_ *_x_*Se nanocrystals or Au nanoparticles, and even the Cu_2−_ *_x_*Se/Au mixture, which was signified by improved tumor‐suppressing efficiency (Figure 24b) and survival rate (Figure 24c) of tumor‐bearing mice. The tumor metastasis into the lung was also decreased by synergistic RT and PTT (Figure 24d). This paradigm provides an efficient strategy for achieving synergistic radiosensitizing and photothermal‐conversion effects by engineering hetero‐nanostructures from high‐ and low‐Z elements.

In addition, ultrasmall CuS nanoparticles was decorated onto the surface of silica‐coated upconversion nanoparticles (UCNPs) with high Z elements (Yb, Gd, and Er), which achieved photothermal tumor ablation as contributed by CuS component and synergistic radiosensitization as contributed by high Z elements in UCNPs.^[^ ^204^ ^]^ Based on the desirable synergy, the tumor was completely eradicated without recurrence for a long duration of 120 days. O_2_‐saturated perfluoropentane (PFP) were delivered by CuS‐decorated hollow MONs for alleviating tumor hypoxia and subsequently enhancing the RT efficiency.^[^ ^205^ ^]^ The photothermal effect of CuS nanoparticles synergistically induced the therapeutic outcome of hypoxic radiosensitization on combating U87MG tumors on mice. ^131^I‐doped CuS nanoparticles (designated as CuS/[^131^I]I) were constructed for synergistic PTT and RT based on the intrinsic photothermal‐conversion effect of CuS nanoparticles and ^131^I radioactivity, yielding the suppression of primary solid tumor, inhibition of tumor metastasis, and prolonging of animal survival.^[^ ^206^ ^]^

**Figure 25:**
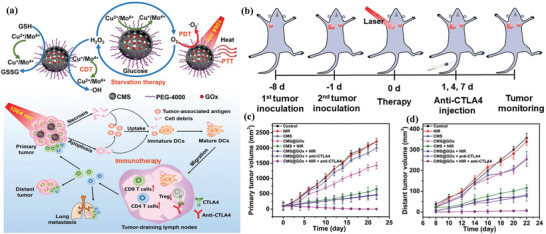
a) The scheme of the underlying therapeutic mechanism on synergistic PTT/PDT/CDT/starvation therapy as enabled by PEGylated CMS@GO*_x_* nanoagents, and the further antitumor immune responses by CMS@GO*_x_*‐enabled multiple nanotherapeutics combined with checkpoint blockade therapy. b) Therapeutic schedule of CMS@GO*_x_*‐enabled synergistic tumor therapy in combination with anti‐CTLA4 checkpoint blockade immune therapy on bilateral tumor models. The tumor‐volume changes of c) primary tumor and d) distant tumor after varied treatments as indicated in the figure. Reproduced with permission.^[^ ^207^ ^]^ Copyright 2019, Wiley‐VCH.

The rational integration of PTT and immunotherapy not only inhibits the primary tumor growth but also suppresses the distant tumor growth by activating the host antitumor immunity.^[^ ^208^, ^209^, ^210^, ^211^, ^212^ ^]^ For instance, chitosan‐coated hollow CuS nanoparticles assembled with immunoadjuvants oligodeoxynucleotides containing the cytosine‐guanine (CpG) motifs for synergistic photothermal immunotherapy against breast cancer on mouse model.^[^ ^213^ ^]^ The typical photothermal effect of CuS component not only inhibited the primary tumor growth, but also released the tumor antigens, and the immunoadjuvants initiated the host antitumor immunity to suppress the distant untreated tumor growth. Glucose oxidase (GO*_x_*)‐loaded hollow mesoporous Cu_2_MoS_4_ (CMS) nanoparticles were constructed for multiple and synergistic cancer therapies relying on their specific reactivity and photonic response.^[^ ^207^ ^]^ First, the presence of multivalent components of Cu(I)/Cu(II) and Mo(IV)/Mo(VI) as nanocatalysts triggered in situ Fenton reaction for producing abundant hydroxyl (•OH) radicals to realize CDT. These CMS nanoparticles also acted as the catalase‐mimicking nanoenzymes to convert endogenous H_2_O_2_ into O_2_, which was further used by the loaded GO*_x_* for converting tumor‐uptaken glucose into H_2_O_2_ for starvation therapy. The specific photonic response of CMS nanoparticles accomplished photothermal ablation (PTT) and PDT of tumors by respectively producing heat and superoxide anion (**Figure** **25**a). Finally, this CMS@GO*_x_*‐based synergistic therapy was developed for rational combination with checkpoint blockade immune therapy (Figure 25b). The use of checkpoint blockade of anticytotoxic T‐lymphocyte antigen‐4 (CTLA4) with the 1064 nm laser‐irradiated CMS@GO*_x_* nanoparticles not only suppressed the primary tumor growth (Figure 25c) but also inhibited the distant tumor growth (Figure 25d), signifying the high synergistic performance on the treatment of metastasis tumors.^[^ ^207^ ^]^ In addition, Cu_2−_ *_x_*Te nanoparticles were demonstrated to mimic both glutathione oxidase and peroxidase under the NIR‐II laser activation, which elevated the oxidative stress in tumor tissue by corresponding GSH depletion and radicals generation, inducing the immunogenic cell death.^[^ ^214^ ^]^ Such intratumor oxidative stress modulated the immunosuppressive microenvironment of the tumor for immunotherapy against tumor metastasis and recurrence, in accompany with the inhibition of primary tumor growth as demonstrated in 4T1 tumor‐bearing BALB/c mice. In addition to the aforementioned typical synergistic strategies, the rational construction of calcium phosphate (CaP)‐doped hollow mesoporous copper sulfide nanoparticles acted as the Ca^2+^ nanogenerator to disrupt the mitochondrial Ca^2+^ homeostasis for inducing cell apoptosis, synergistically enhancing the efficacy of photothermal ablation on combating MCF‐7 breast tumors.^[^ ^215^ ^]^

## 8 Cu‐Involved Contrast Agents for Bioimaging

PA imaging is emerging as a new noninvasive bioimaging technique based on the detection of ultrasound waves as produced by the light absorption‐triggered thermally expanded tissues.^[^ ^216^, ^217^, ^218^, ^219^, ^220^, ^221^ ^]^ PA imaging features distinctive merits as compared to traditional fluorescence imaging, attributing to its enhanced tissue‐penetrating capability. Because the NIR‐responsive photothermal nanoagents can induce the tissue expansion by thermal effect, abundant photothermal nanoagents have been developed as the contrast agents for PA bioimaging. On this ground, photothermal Cu‐involved nanoagents have been extensively explored in PA imaging of tumors in diverse formulations or nanocomposites.^[^ ^222^, ^223^ ^]^ In addition, the distinctive therapeutic capability of Cu‐based nanosystems endows them with PA imaging‐guided therapeutic performances.

**Figure 26:**
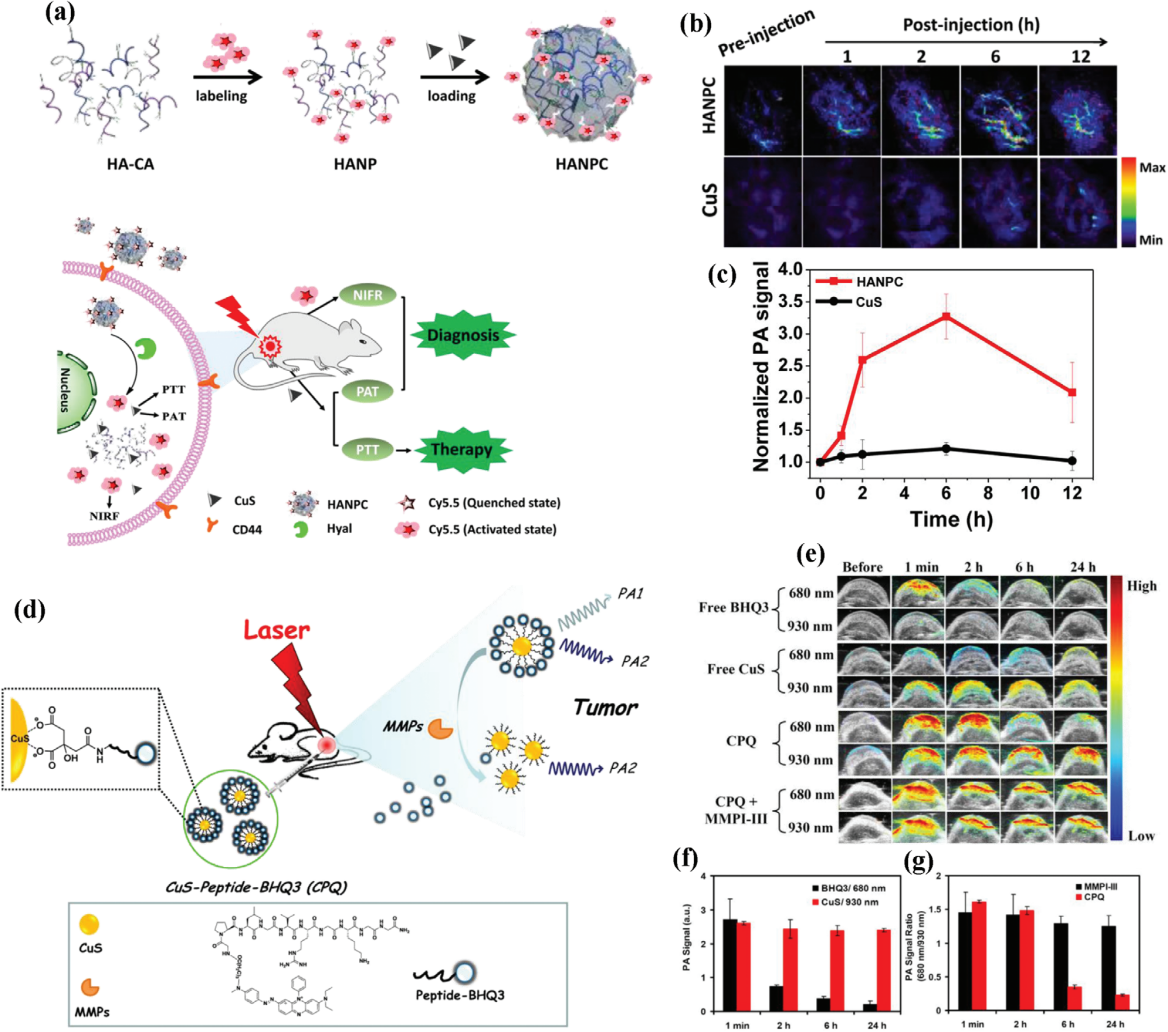
a) Schematic illustration on the construction of targeted PA contrast agent HANPC and their in vivo theranostic performance for PAT/NIRF‐guided PTT. b) In vivo PA imaging of blood vessels on SCC7 tumor‐bearing mice with prolonged observation time after intravenous administration of either HANPC or free CuS, and c) corresponding signal variations. Reproduced with permission.^[^ ^224^ ^]^ Copyright 2014, American Chemical Society. d) The underlying mechanism of CPQ nanoprobe as MMP‐activatable contrast agents of PA imaging. The MMPs broke the MMP‐cleavable peptide linker that was present on the conjugation of BHQ3 fluorescence molecule and CuS nanoparticles. e) In vivo PA imaging of tumor‐bearing mice after the administration of free BHQ3, free CuS, CPQ nanoprobe, and CPQ combined with MMPI‐III. f) PA signal change of the free BHQ3 at 680 nm and free CuS at 930 nm. g) PA signal ratio (680 nm/930 nm) changes of the CPQ group and CPQ + MMPI‐III group with prolonged durations. Reproduced with permission.^[^ ^225^ ^]^ Copyright 2014, Ivyspring.

Based on the targeting capability of HA and photothermal‐conversion performance of CuS nanoparticles, a multifunctional PA contrast agent was fabricated by loading CuS into Cy5.5‐conjugated HA nanoparticles (abbreviated as HANPC) for achieving targeting PA imaging of tumor, in accompany with sensitive fluorescence imaging and photothermal tumor ablation (**Figure** **26**a).^[^ ^224^ ^]^ After the intravenous administration into SCC7 tumor‐bearing mice, a gradual increase of PA signal was observed within the blood of tumor (Figure 26b), where the much‐enhanced PA signal was observed in HANPC group as compared to the free CuS group (Figure 26c) because of the targeting effect of HA component in HANPC. In addition, CuS nanoparticles were developed as the nanoprobes for activatable PA imaging of cancer‐correlated matrix metalloproteinases (MMPs).^[^ ^225^ ^]^ The red‐light‐absorbing organic dye BHQ3 was conjugated with CuS nanoparticles by a cleavable peptide (Figure 26d) to fabricate CuS‐peptide‐BHQ3 (CPQ) agent, which exhibited strong PA signal at 680 and 930 nm. The presence of MMPs in tumor condition induced the release of BHQ3 by breaking the peptide linker, which was quickly excreted out of the tumor, inducing the changes of PA signal ratios of 680 nm/930 nm (Figure 26e–g). Especially, the co‐administration of CPQ nanoprobe and MMP inhibitor‐III caused no profound changes of such a PA signal ratio, signifying the function of MMP for changing the PA signal ratio, which therefore could act as an in vivo indicator for indirectly revealing the MMP activity in tumor microenvironment.^[^ ^225^ ^]^ CuS nanoparticles could also generate PA imaging signals at a wavelength of 1064 nm, potentiating the bioimaging of mouse brain by intracranial injection and rat lymph nodes (12 mm below the skin) after interstitial administration.^[^ ^226^ ^]^ The construction of heterogeneous nanoparticles of Cu_2−_ *_x_*Se and Au accomplished broad LSPR with PA contrast at different wavelengths and maximum PA imaging depth of 17 mm.^[^ ^227^ ^]^

**Figure 27:**
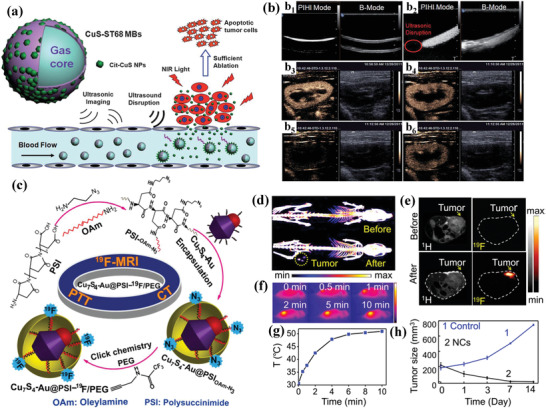
a) The scheme of the theranostic functionality of CuS‐ST68 MBs for ultrasound imaging and UTMD‐induced photothermal ablation. b) In vitro ultrasound imaging in a latex tube b_1_) in the presence and b_2_) in the absence of CuS‐ST68 MBs under PIHI mode and B mode. In vivo ultrasound imaging of rabbit right kidney after the administration of b_3_) ST68 MBs and b_4_) CuS‐ST68 MBs. b_5_) Ultrasound imaging of rabbit right kidney after focused ultrasonic disruption of CuS‐ST68 MBs and b_6_) reperfusion of CuS‐ST68 MBs into kidneys for ultrasound imaging. Reproduced with permission.^[^ ^228^ ^]^ Copyright 2013, Royal Society of Chemistry. c) The scheme on the construction of Cu_7_S_4_–Au@PSI‐^19^F/PEG nanoparticles for CT and ^19^F‐MR dual‐modality bioimaging and photothermal tumor hyperthermia. d) CT imaging of tumor‐bearing mice before and after the administration of nanoprobes, and e) the ^1^H‐ and ^19^F‐MR imaging of tumor‐bearing mice after the nanoprobes injection. f) The thermal images of tumor‐bearing mice after the administration of composite nanoagents followed by 808 nm laser irradiation, and g) the corresponding temperature‐elevating curve. h) The tumor size curves after the photothermal hyperthermia with prolonged feeding durations. Reproduced with permission.^[^ ^229^ ^]^ Copyright 2018, American Chemical Society.

Ultrasound imaging is featured with noninvasiveness, convenience, portability and cost‐effectiveness, which has been broadly used in clinical biomedicine.^[^ ^24^, ^230^, ^231^, ^232^ ^]^ The rational combination of Cu‐involved nanoparticles with contrast agents of ultrasound imaging can produce ultrasound‐responsive theranostic nanoagents for satisfying varied biomedical application requirements. On this ground, CuS nanoparticles were decorated onto the surface of perfluoropropane gas‐filled microbubbles (MBs) that were fabricated by a micellar solution with the composition of Span 60 and Tween 80 (abbreviated as CuS‐ST68 MBs; **Figure** **27**a).^[^ ^228^ ^]^ The constructed CuS‐ST68 MBs not only exerted the functionality for ultrasound imaging (Figure 27b), but also released the loaded CuS nanoparticles for photothermal ablation after the ultrasound‐triggered microbubble destruction (UTMD). The in vivo administration of CuS‐ST68 MBs induced obvious contrast enhancement in the rabbit kidney, signifying the high potential for ultrasound imaging. However, the large particle size of micrometer‐sized microbubbles can only be present in the blood vessels for blood‐pool bioimaging. To solve this critical issue, laser‐activated perfluorocarbon nanodroplets with nanometer sizes (250 nm) were combined with CuS nanoparticles with the specific capability of both PA and US bioimaging.^[^ ^233^ ^]^ Their small particle size guaranteed efficient penetration into tumor tissue. The presence of CuS nanoparticles vaporized perfluorocarbon nanodroplets to produce gaseous microbubbles, efficiently making the contrast enhancement in US imaging, in accompany with the intrinsic PA‐imaging performance of the CuS component.

Gd‐integrated CuS nanoparticles were synthesized by using BSA as the template (abbreviated as Gd:CuS@BSA). These Gd:CuS@BSA nanoplatforms acted as the contrast agents for dual bioimaging, where the Gd component was used for T_1_‐weighted MR imaging with a high *r*_1_ value of 16.032 mm ^−1^ s^−1^ and CuS functioned as the PA contrast agents.^[^ ^234^ ^]^ Ni‐integrated CuS nanoparticles exhibited positive MRI contrast with enhanced T_1_ relaxivity as compared to Ni ions.^[^ ^235^ ^]^ Similarly, the Fe^3+^ doping into Cu_5_FeS_4_ nanoparticles also guaranteed the contrast‐enhanced T_1_‐weighted MR imaging.^[^ ^236^ ^]^ In addition, Au domain was grown on to a Cu_7_S_4_ domain to produce Cu_7_S_4_–Au heterodimers, which were further grafted with ^19^F‐MRI signal molecules (Figure 27c).^[^ ^229^ ^]^ The generated Cu_7_S_4_–Au@PSI‐^19^F/PEG nanocomposites acted as dual‐modality contrast agents for both CT imaging and ^19^F‐MR imaging. On tumor‐bearing mice, the administration of Cu_7_S_4_–Au@PSI‐^19^F/PEG nanoprobes exhibited contrast‐enhanced CT imaging with high HU values (Figure 27d).^[^ ^229^ ^]^ Especially, the boundary between tumor and normal tissue was delineated clearly by ^19^F MR imaging based on the high signal‐to‐noise ratio originating from negligible endogenous background influence, which was comparatively difficult to be distinguished in traditional ^1^H‐MR imaging (Figure 27e). The photothermal effect of Cu_7_S_4_–Au heterodimers was effective in NIR‐induced temperature elevation in the tumor (Figure 27f,g), inducing the high tumor‐suppressing outcome based on the photothermal tumor hyperthermia (Figure 27h).

Positron‐emitting isotope ^64^Cu‐labeled CuS nanodot‐decorated MSNs exhibited contrast‐enhanced micro‐PET/CT imaging of tumor after intravenous administration for revealing the underlying pharmacokinetics and biodistributions, demonstrating the efficient and continuous tumor accumulation.^[^ ^237^ ^]^ Similarly, ^64^Cu‐labeled CuS@MSN with surface TRC105 monoclonal antibody was employed for in vivo targeted PET imaging of tumor vasculature.^[^ ^238^ ^] 64^Cu‐labeled ultrasmall CuS nanodots exhibited high excretion by renal clearance,^[^ ^239^ ^]^ providing the high potential for PET imaging‐guidance photothermal tumor ablation.^[^ ^240^, ^241^ ^]^ The targeted PET imaging was also realized by introducing tumor‐targeting ligand folic acid (FA) onto the surface of ^64^Cu‐labeled CuS nanoparticles against folate‐receptor‐expressing KB tumor xenograft on mice.^[^ ^242^ ^]^

**Figure 28:**
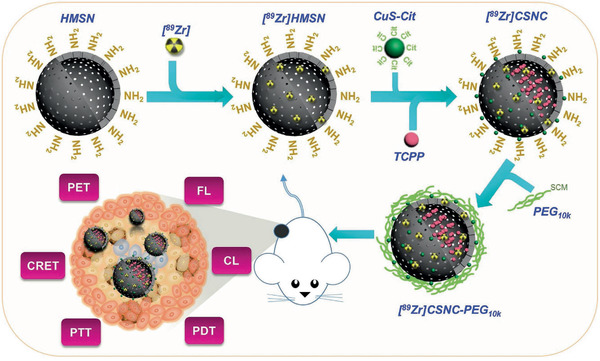
The scheme on the construction of radiolabeled core‐satellite nanoconstructs (CSNCs) for tetra‐modality bioimaging (PET, FL, CL, and CRET imaging) and synergistic PTT and PDT. Reproduced with permission.^[^ ^243^ ^]^ Copyright 2018, Wiley‐VCH.

The integration of CuS nanoparticles onto the surface of [^89^Zr]‐labeled hollow mesoporous silica nanoparticles with the loaded meso‐TCPP was rationally designed to construct a theranostic nanoagent with tetra‐modality imaging and synergistic PTT/PDT therapeutic performance (**Figure** **28**).^[^ ^243^ ^]^ The obtained radiolabeled core‐satellite nanoconstructs (CSNCs) were employed for concurrent PET, fluorescence (FL), Cerenkov luminescence (CL), and Cerenkov resonance energy transfer (CRET) imaging of 4T1 tumor‐bearing mice. The labeled zirconium‐89 (^89^Zr, *t*_1/2_ = 78.4 h) was used for PET, CL, and CRET imaging. The loaded TCPP not only acted as the contrast agents for FL imaging, but also functioned as the photosensitizer for photodynamic tumor therapy. The integrated CuS nanoparticles converted NIR light into thermal energy for synergistically enhancing the PDT efficacy on tumor eradication. This paradigm signified that the rational combination of Cu‐involved nanoagents with functional moieties can not only achieve multimodality bioimaging, but also realize multimodality nanotherapy. In addition to above‐mentioned diagnostic‐bioimaging modalities, the rational integration of Cu‐involved nanosystems with fluorescent components could endow them with luminescence‐bioimaging property, such as the in situ growth of CuS onto NaYF_4_:Yb,Er@NaYF_4_:Nd,Yb UCNP nanoparticles for 808 nm laser‐activated green luminescence‐guided photothermal ablation.^[^ ^244^ ^]^

## 9 Biological Effect and Biosafety of Cu‐Involved Nanosystems

**Figure 29:**
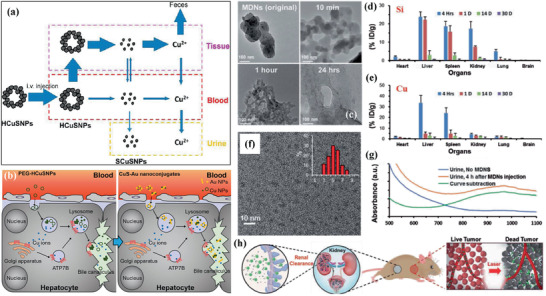
a) Schematic illustration of the in vivo structure/composition evolution of HCuSNPs and further excretion out of the body. Reproduced with permission.^[^ ^47^ ^]^ Copyright 2013, American Chemical Society. b) Schematic illustration of the underlying mechanism on the hepatobiliary excretion of PEGylated HCuSNPs (left scheme) and CuS–Au nanoconjugates (right image). Reproduced with permission.^[^ ^245^ ^]^ Copyright 2019, American Chemical Society. c) TEM images of MDNs after the irradiation of NIR laser in PBS with prolonged durations. Biodistribution of d) Si and e) Cu in major organs after the intravenous administration of MDNs. f) TEM image and g) UV–vis spectra of urine after the intravenous administration of MDNs for 4 h. h) Schematic illustration of the clearance of MDNs after intravenous administration. Reproduced with permission.^[^ ^207^ ^]^ Copyright 2018, Wiley‐VCH.

Copper (Cu) is an important trace element to maintain the health of living creatures with the highest safe intake amount of around 10 mg per day in adults.^[^ ^47^ ^]^ Especially, copper deficiency can induce a series of diseases including cardiovascular disease and diabetes.^[^ ^246^, ^247^, ^248^, ^249^ ^]^ However, the high Cu accumulation might induce the potential toxicity issue in the body, despite some Cu‐based nanoagents have been preliminarily demonstrated to be biocompatible. Therefore, the in vivo biological effect and biosafety of the developed Cu‐involved nanosystems should be carefully and systematically assessed for guaranteeing their further clinical translation. For instance, the in vivo structure/composition evolution and further excretion of hollow CuS nanoparticles (abbreviated as HCuSNPs) were revealed in detail.^[^ ^47^ ^]^ After intravenous administration, these PEGylated HCuSNPs gradually degraded into small‐sized CuS nanoparticles, which then degraded into Cu ions (**Figure** **29**a). The excretion of these HCuSNPs was based on the postgenerated small‐sized CuS nanoparticles and Cu ions, which were readily cleared by both hepatobiliary and renal excretion. Based on the easy biodegradation of MOF‐based nanosystems, the Cu‐composed CuTz‐1‐O_2_@F127 MOFs exhibited initial high accumulation in the liver and spleen.^[^ ^107^ ^]^ However, these nanosized MOFs degraded gradually with the excretion out of the body via feces and urine, by which about 90% nanoparticles were discovered to be excreted out of the body to guarantee the high biosafety of these MOFs. In addition, it has been demonstrated that the extraction of PEGylated HCuSNPs was strongly correlated to the P‐type ATPase Cu transporter ATP7B, which could mediate the exocytosis of nanosized CuS nanoagents to induce the quick hepatobiliary excretion (Figure 29b).^[^ ^245^ ^]^ The as‐established ATP7B‐mediated hepatobiliary CuS excretion was further used to augment the metabolism of Au nanoparticles out of the body based on the construction of CuS–Au nanoconjugates, which was signified by the combination of 80 nm sized HCuSNPs with 40 nm sized Au nanoparticles and integration of 5 nm sized CuS nanoparticles with Au nanorods (length: 40 nm; width: 10 nm).

The photothermal effect of CuS nanoparticles triggered the fast biodegradation of CuS nanodot‐decorated and DOX‐laden MSNs (abbreviated as MDNs) in PBS solution with prolonged durations (Figure 29c). The biodistribution analysis revealed the presence of Si and Cu elements in the kidney, signifying the quick clearance of nanoparticles by the renal‐urinary system after the biodegradation in the body (Figure 29d,e). The presence of ultrasmall CuS nanodots in the urine and corresponding characteristic UV–vis spectrum of CuS nanodots further demonstrated the biodegradation of MDNs and renal excretion (Figure 29f,g). Taking these results together, these multifunctional MDNs efficient accumulated into tumor for nanotherapeutic purposes, and they could be gradually biodegraded into small fragments with the subsequent renal clearance out of the body (Figure 29h). Ultrasmall CuS nanoparticles could be directly excreted by renal‐urinary system where nearly 95% amount was cleared within 24 h.^[^ ^239^ ^]^

The surface engineering Cu‐involved nanosystems enable the active targeting of these nanoparticles into lesion tissues. For instance, the surface conjugation of CuS‐based micelles with GE11 peptides targeted to the triple‐negative breast cancer with overexpressed epidermal growth factor receptor.^[^ ^201^ ^]^ The receptor‐mediated targeting endocytosis was enabled by folic acid conjugation onto the surface of hollow MSNs with surface‐anchored CuS nanoparticles.^[^ ^199^ ^]^ TRC105, as the human/murine chimeric IgG1 monoclonal antibody, was conjugated onto the surface of ^64^Cu‐labeled CuS@MSN nanoparticles for targeted PET imaging of tumor vasculature based on its binding capability to CD105 on tumor neovasculature.^[^ ^238^ ^]^ Similarly, FA was employed for surface conjugation of CuS nanoparticles for targeting folate receptor‐expressing KB tumors.^[^ ^242^ ^]^ Despite the excretion of some nanosystems that have been revealed, the biodistribution and kinetics of the administrated Cu‐involved nanoparticles in vivo are still unclear, which might also vary in different animal models. The underlying biological effect should be assessed and revealed at the fundamental level, where the hazard/risk assessment is still highly lacking at current stage.

## 10 Conclusions and Outlook

As one of the most representative transitional metal elements, copper (Cu)‐involved nanosystems have attracted ever‐increasing attention of the scientific community in pharmacy and biomedicine, not only because of their readily modulated nanostructures and versatile compositions, but also due to their unique physiochemical attributes and biological effects, endowing them with high theranostic performance in biomedicine. It is traditionally regarded that, as the transitional metal elements, Cu ions are more toxic as compared to Fe ions or Mn ions, therefore the biomedical use of Cu ions in biomedicine is relatively limited. Fortunately, the fabricated Cu‐involved nanoparticles have been demonstrated to be nontoxic with high biocompatibility and biosafety, solving the critical toxicity issue of Cu use in biomedicine. It is noted that the prerelease of Cu ions from Cu‐based nanosystems should be avoided before exerting the theranostic performance to avoid the potential toxicities. In addition, these rationally designed Cu nanosystems exert the fascinating attributes in theranostic nanomedicine that are not possessed in Cu ions, such as the photothermal or photodynamic effects. The conversion of Cu ions into Cu nanoparticles also altered the in vivo biological behaviors of Cu species, rendering them more biocompatible and biosafe for guaranteeing the potential clinical translation. On this ground, as highlighted in detail above, Cu‐involved nanosystems have been extensively exploited in numerous biomedical applications, such as tumor therapy, antibacteria, tissue‐regeneration, and bioimaging, which are strongly dependent on their physicochemical properties and biological effects, including photothermal/photodynamic effect, catalytic property, bone/skin tissue‐regenerating bioactivity, antibacterial behavior, PA‐imaging capability, etc.

Versatile Cu‐composed nanosystems have been discussed and summarized in this review. Typically, these Cu‐involved nanosystems include organic, inorganic, or organic–inorganic hybrid nanoplatforms. Cu‐composed organic systems generally feature high biocompatibility and desirable biodegradability. Comparatively, the biological effect and biosafety of inorganic nanosystems have not fully revealed, showing the difficulty in modulating and optimizing the biocompatibility and biodegradability. However, the inorganic nanosystems typically possess more functionalities (optical, acoustic, electrical, and magnetic property) for developing some distinctive theranostic modalities as compared to organic nanoparticles. The organic–inorganic hybrid nanosystems can combine the advantages of organic and inorganic nanosystems, showing high promise for further clinical translations. In addition, the precise modulation of the valence of Cu components in these nanoparticles can efficiently control the photoresponsive behavior and catalytic activity, which can substantially enhance their therapeutic performance. The design originality of these Cu‐involved nanosystems strongly depends on the practical application requirements because different biomedical uses usually need the corresponding functionalities and biological effects.

**Figure 30:**
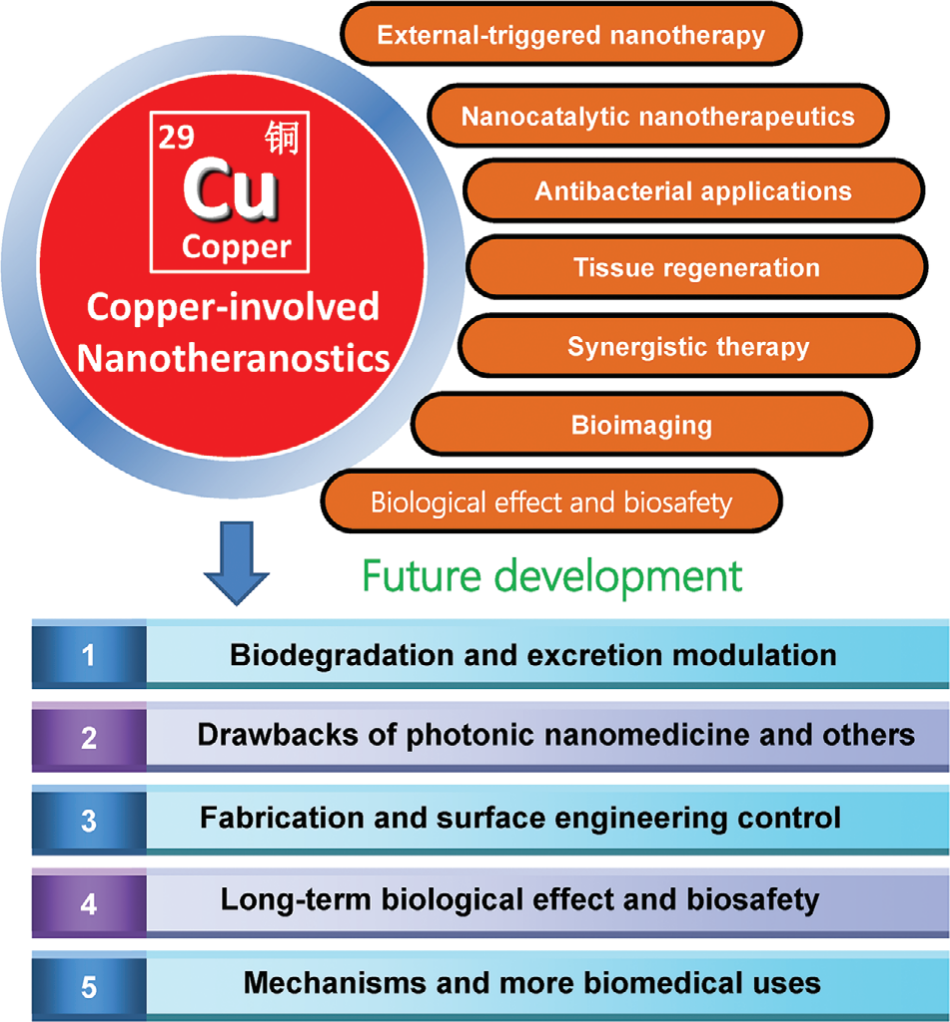
Summative scheme of the current progresses and future developments of copper‐involved nanotherapeutics.

It is noted that most of the progresses of Cu‐involved nanomedicine have been made in the past ten years. The new biomedical use in catalytic medicine only emerges very recently. Therefore, this Cu‐correlated theranostic nanomedicine field is till at the preliminary stage, in together with several unresolved critical issues and facing hurdles during their further clinical translations, such as the biodegradation issue, photoresponse drawback, difficult fabrication, structural/composition modulation and surface engineering, and long‐term biological effect and biosafety (**Figure** **30**).

The first consideration is the biodegradation of most Cu‐involved nanosystems, which are mostly in the formula of inorganic composition with a low biodegradation rate. Compared to traditional organic nanosystems with desirable biodegradability, the biodegradation of inorganic nanoparticles is still under debate, causing their accumulation within the body and difficulty in excretion. Most of these inorganic Cu nanoparticles have been demonstrated to be excreted and cleared by feces and urine in the form of intact nanoparticles. However, because of the low biodegradation rate, the remaining nanoparticles in the body with unclear long‐term fate might induce the adverse effect and potential toxicity, which has not been revealed and demonstrated. One possible strategy to solve this critical issue is the rational modulation of dispersity, particle size and surface modification for expediting their excretion rate and amount by feces and urine, minimizing the side effects of nonbiodegraded Cu nanoparticles on the body. The other methodology is attempted to design biodegradable Cu‐involved nanosystems depending on the advances of nanosynthetic chemistry and nanomaterial science, such as the demonstrated Cu‐doped MSNs and Cu‐based MOFs.

The second consideration is the photoresponse of some Cu nanosystems for photonic nanomedical applications. Although photonic nanomedicine has gotten ever‐increasing progresses in recent years, the low tissue‐penetrating depth of light severely hinders the theranostics of deep‐seated diseases, which means that they can only treat the superficial diseases such as skin diseases. The same shortcoming is also present in Cu nanosystem‐based photonic nanomedicine. The precise modulation of the nanostructure, composition, and physiochemical property has been demonstrated to strengthen the photothermal‐conversion efficiency and extend the photoresponsive wavelength into the second NIR biowindow, which can efficiently enhance the tissue‐penetrating depth of light. In addition, the integration of photonic nanotherapy with other therapeutic modalities can achieve the synergistic therapeutic outcome, further overcoming the low therapeutic efficiency of phototherapies originating from the low tissue‐penetrating capability of light.

The third consideration is the large‐scale fabrication and surface‐engineering issues of Cu nanoparticles for satisfying the clinical translation requirements. Compared to the mostly exploited traditional metal Fe‐ or Mn‐based nanoparticles, most of Cu‐involved nanosystems emerge in recent years, indicating that their synthetic methodologies are not mature, making the structure and composition modulation difficult. It is well‐known that the industrial translation and biomedical use require large‐scale and repeatable production of nanomedicines with high repeatability. Obviously, the current synthetic strategies cannot meet this requirement where the production should be upscaled to an industrial scale. The current construction of Cu nanoparticles predominantly focuses on the revealing of their theranostic performance. Less attention has been paid on their synthetic methodologies with translational potentials, where the expensive reagents were used and the approaches were difficult to be scaled up. This issue is expected to be resolved by the advances of nanosynthetic chemistry and material science. In addition, the surface engineering of Cu nanoparticles is of high significance for determining their in vivo behaviors and performances, such as long blood‐circulation duration and high tumor‐targeted accumulation. The surface modification and engineering of Cu‐involved nanosystems strongly depend on the synthetic strategies and final products because bare inorganic Cu nanoparticles do not have useful groups for chemical conjugation, which generally requires the fabrication of Cu‐based nanocomposites, making the final nanosystems relatively complex and therefore difficult for further clinical translation. It should be noted that “elaborate formulations” with multiple compositions is not suggested because the too much complexity usually causes the insufficient reproducibility.

The fourth consideration is the long‐term biological effects and biosafety of these Cu‐involved nanosystems those have not been assessed and revealed yet. The available biosafety data typically concentrate on the relatively short‐term biosafety evaluation of usually less than one month. Almost all reports declare the high biosafety of the developed Cu nanoparticles within the adopted periods and doses, which however cannot guarantee the long‐term biosafety because of the lack of solid evidence. Therefore, the standard principles and methodologies on toxicity and biosafety evaluations of these Cu‐based nanoparticles should be adopted for guaranteeing their further clinical translations rather than the simple and preliminary short‐term assessment with limited data to declare the high biosafety of these Cu nanosystems.

The fifth consideration is the thorough mechanism investigation and more exploration of biomedical use of these emerging Cu‐based nanosystems. It has been discovered that most of the publications primarily report on the theranostic performance on combating diverse diseases. The underlying chemical, physical and biological mechanisms have not been fully studied and clarified, which should be revealed at the fundamental level. Of source the performance is important, but the fundamental scientific concerns are also of equivalent significance because they can inspire the researchers with possible optimizing strategies for strengthening the theranostic performances in the following researches. In addition, as compared to traditional Fe/Mn‐based nanomedicines, Cu‐involved nanosystems are still in the infancy for biomedical use, indicating that they have more properties and applications to be exploited in the future developments, besides the aforementioned several representative biomedical aspects.

By entering the “Coppery Age,” abundant Cu‐involved nanosystems have been preliminarily explored in versatile biomedical applications. Moving forward, these as‐demonstrated theranostic performances encourage us to further explore their practical applications in clinic. The following researches should focus more on the biological effects of introducing these Cu species in the form of nanoparticles into the body. It is also highly suggested to standardize new drug‐development protocols, methods and principles, not only for promoting the clinical translations of available numerous Cu nanoparticles but also for creating new emerging Cu‐involved nanosystems in theranostic nanomedicine, by which the experimental results from different groups and labs can be compared and promoted with each other with desirable data traceability.

**Table 1:** Selected paradigms of copper‐involved nanotheranostics for versatile biomedical applications

Num.	Cu‐involved nanosystems	Nanotheranostic modality	Nanotheranostic performance	Refs.
1	Cu_2−_ *_x_*S	Photothermal therapy	Featuring high photothermal‐conversion efficiency of 64.8% at 808 nm for substantially inhibiting tumor growth with no reoccurrence and metastasis.	^[^ ^67^ ^]^
2	Fe_3_O_4_@CuS	Photothermal therapy	Photonic response in the second NIR biowindow (1064 nm) for magnetic‐targeted photothermal tumor ablation.	^[^ ^71^ ^]^
3	Cu_9_S_5_	Photothermal therapy	Possessing improved absorption (1.2 × 109 m ^−1^ cm^−1^) and photothermal‐conversion efficiency (25.7%) at 980 nm.	^[^ ^72^ ^]^
4	CuS‐ZIF‐8	Photothermal therapy and chemotherapy	NIR‐induced dissociation of ZIF‐8 for the release of loaded chemotherapeutic drug, inducing synergistic photothermal ablation and NIR‐triggered chemotherapy.	^[^ ^75^ ^]^
5	Fe^3+^‐Cu_2−_ *_x_*Se	Photothermal therapy and MR imaging	Ferric ions (Fe^3+^) for modulating the vacancy of Cu_2−_ *_x_*Se nanoparticles with adaptable NIR absorption and MRI performance.	^[^ ^76^ ^]^
6	Au@Cu_2−_ *_x_*S	Photothermal therapy	High photothermal‐conversion efficiency of 59% at 808 nm and 43% at 1064 nm because of the coupled LSPR properties of Au and Cu_2−_ *_x_*S.	^[^ ^78^ ^]^
7	Cu_2_O	Photothermal therapy and PA imaging	Overexpressed endogenous H_2_S in colon cancer in situ converting Cu_2_O into copper sulfide for activatable PA imaging and photothermal tumor ablation.	^[^ ^81^ ^]^
8	Au@Cu_2_O	Photothermal therapy and PA imaging	Overexpressed endogenous H_2_S in colon cancer in situ converting Au@Cu_2_O into Au@Cu_9_S_8_ for activatable and enhanced PA imaging and photothermal tumor ablation.	^[^ ^82^ ^]^
9	Ce6/Cu_2−_ *_x_*S	Photothermal therapy and photodynamic therapy	Mitochondrial‐targeted delivery and synergistic PTT/PDT on synergistically combating tumor.	^[^ ^91^ ^]^
10	Fe*_x_*Cu*_y_*Se	Photothermal therapy	Protecting the A*β*42‐induced neuronal damage and mitigating the symptoms in AD mouse model.	^[^ ^95^ ^]^
11	Cu‐Cys	Catalytic therapy	Achieving GSH‐responsive chemodynamic tumor therapy	^[^ ^106^ ^]^
12	CuTz‐1@F127	Catalytic therapy and photodynamic therapy	Generating Fenton‐like reaction‐based hydroxyl radical (•OH) and photodynamic effect‐induced singlet oxygen (^1^O_2_) for synergistic tumor therapy.	^[^ ^107^ ^]^
13	CuO_2_	Catalytic therapy	H_2_O_2_ self‐supplying catalytic therapy for suppressing tumor growth.	^[^ ^108^ ^]^
14	Cu^2+^‐C_3_N_4_	Catalytic therapy and photodynamic therapy	Featuring synergy on enhancing g‐C_3_N_4_‐based PDT efficacy by copper(II) coordination and the intrinsic catalytic performance of Cu ions.	^[^ ^110^ ^]^
15	Cu‐TCPP	Catalytic therapy	Cu‐TCPP for achieving ^1^O_2_ production independent on the oxygen and external light irradiation.	^[^ ^111^ ^]^
16	Cu*_x_*Co*_y_*S	Photodynamic therapy	Phototriggered intracellular large ROS production ROS for tumor therapy.	^[^ ^112^ ^]^
17	Cu_2_(OH)PO_4_	Catalytic therapy	X‐ray irradiation for converting Cu^II^ sites into Cu^I^ sites with enhanced catalytic activities in triggering Fenton reaction and high tumor‐suppressing efficacy.	^[^ ^94^ ^]^
18	G‐CuS‐DOX	Drug delivery and photothermal therapy	Achieving synergistic photothermal ablation and photocontrollable chemotherapy with high tumor‐suppression efficacy.	^[^ ^117^ ^]^
19	CuS@Cu_2_S@Au	Drug delivery and photothermal therapy	Featuring photoswitchable targeting transportation and targeted chemotherapy combined with the enhanced photothermal ablation.	^[^ ^119^ ^]^
20	HMCuS	Drug delivery and photothermal therapy	Resulting in synergistic chemotherapy and photothermal hyperthermia against tumor growth.	^[^ ^120^ ^]^
21	CuS@PMO	Drug delivery and photothermal therapy	CuS‐enabled hyperthermia effect for enhancing the intracellular uptake of CuS@PMO and strengthening the synergistic therapeutic efficacy of chemotherapy and photothermal ablation.	^[^ ^127^ ^]^
22	Cu‐HMSNs	Drug delivery	Enabling the release of loaded DSF and Cu ions in Cu‐HMSNs for enhanced DSF chemotherapy.	^[^ ^129^ ^]^
23	CuI	Antibacteria	Producing ROS in both gram‐negative and gram‐positive bacteria for inducing antibacterial activity by DNA and membrane damage.	^[^ ^161^ ^]^
24	CuO	Antibacteria	Featuring shape‐dependent antibacterial activity where the plate‐like CuO exhibited higher antibacterial performance as compared to grain or needle shaped CuO nanomaterials.	^[^ ^163^ ^]^
25	Cu‐MBGs	Antibacteria	Exhibiting desirable antibacterial effect on killing *E. coli*, *S. aureus*, and *S. epidermidis*, and further disrupting the *S. epidermidis*‐induced biofilm dispersion.	^[^ ^167^ ^]^
26	CuO‐HCSs	Antibacteria	Cu‐HCSs nanoenzymes for inducing peroxidase‐mimicking catalytic reactions to produce ROS, and causing the oxidative damage of both gram‐positive and gram‐negative bacterial.	^[^ ^168^ ^]^
27	AgCu	Antibacteria	Displaying enhanced antibacterial bioactivity against *Escherichia coli* as compared to equivalent Ag nanoparticles.	^[^ ^169^ ^]^
28	Cu_9_S_8_	Antibacteria	Inducing both photothermal and photodynamic effects for destructing bacterial biofilm (*S. aureus*) on the Ti plates.	^[^ ^171^ ^]^
29	Cu‐MBG	Tissue regeneration	Facilitating the wound healing by inducing a profound angiogenic effect, in accompany with the antibacterial bioactivity.	^[^ ^175^ ^]^
30	GO–Cu	Tissue regeneration	Achieving vascularized bone regeneration by graphene for promoting the osteogenic differentiation of BMSCs and Cu‐involved nanoparticles for maintaining the bone volume and accelerating the bone‐healing rate.	^[^ ^177^ ^]^
31	CSO‐integrated scaffolds	Tissue regeneration and cancer therapy	Achieving synergistic chemotherapy and photothermal ablation of melanoma and skin‐tissue regeneration.	^[^ ^179^ ^]^
32	Cu‐MOF	Tissue regeneration	Promoting the angiogenesis and collagen deposition for accelerating the healing of chronic nonhealing wounds in diabetic mice with splinted excisional dermal wound.	^[^ ^181^ ^]^
33	AuAgCu_2_O	Tissue regeneration and antibacteria	Combating drug‐resistant bacterial infection and accelerating the recovery of cutaneous chronic wound and nonhealing keratitis.	^[^ ^182^ ^]^
34	Copper(I) phosphide	Photothermal therapy and catalytic therapy	Inducing photothermal ablation and photothermal‐enhanced Fenton‐like reaction‐induced oxidative cancer therapy.	^[^ ^190^ ^]^
35	Cu_2−_ *_x_*S	Photothermal therapy and catalytic therapy	Synergistic NIR‐II‐induced photothermal therapy and catalytic therapy on suppressing tumor growth.	^[^ ^191^ ^]^
36	Cu_2−_ *_x_*E–Au	Photothermal therapy and radiation therapy	Featuring synergistic photothermal ablation and radiation therapy on tumor‐growth inhibition.	^[^ ^203^ ^]^
37	CuS‐UCNPs	Photothermal therapy and radiation therapy	Achieving photothermal tumor ablation as contributed by CuS component and synergistic radiosensitization as contributed by high Z elements in UCNPs.	^[^ ^204^ ^]^
38	CuS‐CpG	Photothermal therapy and immunotherapy	Photothermal therapy‐induced inhibition of primary tumor growth and immunoadjuvant‐initiated host antitumor immunity for suppressing the distant untreated tumor growth.	^[^ ^213^ ^]^
39	CMS@GO*_x_*	Photothermal therapy and photodynamic therapy and immunotherapy	Achieving synergistic photothermal therapy and photodynamic therapy on combating tumor, with the further checkpoint blockade immune therapy.	^[^ ^207^ ^]^
40	CaP‐copper sulfide	Photothermal therapy and Ca^2+^‐based therapy	Acting as the Ca^2+^ nanogenerator to disrupt the mitochondrial Ca^2+^ homeostasis for inducing cell apoptosis, synergistically enhancing the efficacy of photothermal ablation.	^[^ ^215^ ^]^
41	HA‐CuS	Photoacoustic imaging	Achieving targeting PA imaging of SCC7 tumor on mice.	^[^ ^224^ ^]^
42	CuS‐MBs	Ultrasound imaging	Featuring ultrasound imaging‐guided photothermal tumor ablation.	^[^ ^228^ ^]^
43	Gd:CuS@BSA	Magnetic resonance imaging	Achieving T_1_‐weighted magnetic resonance imaging with high *r*_1_ value of 16.032 mm ^−1^ s^−1^.	^[^ ^234^ ^]^
44	Cu_7_S_4_–Au@PSI‐^19^F/PEG	Magnetic resonance imaging and CT imaging	Exhibiting contrast‐enhanced CT imaging with high HU values. The boundary between tumor and normal tissue was delineated clearly by ^19^F MR imaging based on the high signal‐to‐noise ratio originating from negligible endogenous background influence.	^[^ ^229^ ^]^
45	^64^Cu‐labeled CuS	Positron emission tomography imaging	Revealing the underlying pharmacokinetics and biodistributions and demonstrating the efficient and continuous tumor accumulation.	^[^ ^237^ ^]^
46	^64^Cu‐labeled CuS@MSN	Positron emission tomography imaging	Achieving in vivo targeted PET imaging of tumor vasculature.	^[^ ^238^ ^]^
47	^64^Cu‐labeled CuS	Positron emission tomography imaging	PET imaging‐guidance photothermal tumor ablation with high excretion by renal clearance because of ultrasmall size.	^[^ ^239^ ^]^
48	CSNC	Multiple imaging	Achieving concurrent positron emission tomography (PET), fluorescence (FL), Cerenkov luminescence (CL), and Cerenkov resonance energy transfer (CRET) imaging of tumor.	^[^ ^243^ ^]^
49	HCuSNPs	Biological effect	Their excretion was based on the postgenerated small‐sized CuS nanoparticles and Cu ions, which were readily cleared by both hepatobiliary and renal excretion.	^[^ ^47^ ^]^
50	CuS–Au	Biological effect	Augmenting the metabolism of Au nanoparticles out of the body by ATP7B‐mediated hepatobiliary CuS excretion.	^[^ ^245^ ^]^

## Conflict of Interest

The authors declare no conflict of interest.
